# Cdc42EP3-bound septin scaffolds promote actin polymerization

**DOI:** 10.1016/j.jbc.2025.108325

**Published:** 2025-02-18

**Authors:** Meagan R. Tomasso, Prajakta D. Mehetre, Priyashree Nagarajan, Roshni Ravi, Jennifer Byrnett, Eric Brinckman, Joseph Magliozzi, Bruce L. Goode, Shae B. Padrick

**Affiliations:** 1 Department of Biochemistry and Molecular Biology, Drexel University, Philadelphia, Pennsylvania, USA; 2 Department of Biology, Rosenstiel Basic Medical Science Research Center, Brandeis University, Waltham, Massachusetts, USA

**Keywords:** actin, microfilaments, G-actin, cytoskeleton, septin, Cdc42EP/BORG, CDC42, filament nucleation, bundling

## Abstract

Septins are cytoskeletal filament-forming proteins that typically associate with membranes and perform critical functions in a variety of cellular processes. Septins often colocalize with actin and microtubule structures, yet our understanding of all the ways that septins contribute mechanistically to actin- and microtubule-based functions is incomplete. The Cdc42 effector protein Cdc42EP3 (also known as BORG2) promotes septin localization to actin structures *in vivo*, but little else is known about how Cdc42EP3 influences the interactions of septins and F-actin. Here, using purified components, we show that Cdc42EP3 binds directly to septins, actin filaments, and actin monomers. Moreover, septin-bound Cdc42EP3 accelerates actin filament polymerization. Thus, Cdc42EP3 is not merely a factor that crosslinks septins and F-actin, but one that promotes the formation of actin polymers along septin scaffolds.

The septins are a family of cytoskeleton proteins with cellular roles in focal adhesion maturation, cell division, membrane structure, cell-cell adhesion, and ciliogenesis (1, 2, 3, 4, 5, 6). In addition, septins play a part in multiple pathogenic processes including bacterial infection, neurological diseases, and numerous cancers (2, 7, 8, 9, 10, 11, 12, 13). A key family of septin-interacting proteins, the Cdc42EP or BORG family, has similarly been tied to the progression of multiple cancers, often in the context of septins (14). Thus, uncovering the mechanisms that underlie septin and Cdc42EP/BORG cellular functions may yield new insights into human disease.

There are thirteen septins in mammals, SEPT1-SEPT12 and SEPT14. Based on sequence similarity, these thirteen septins have been further classified into four subgroups: SEPT2, SEPT6, SEPT7, and SEPT3 (15, 16). Septins of different subgroups hetero-oligomerize to form hexameric complexes containing septins from subgroups SEPT2, SEPT6, and SEPT7, and octameric complexes that also contain SEPT3 subgroup members, such as SEPT9 (17, 18, 19). Biochemically, the best-studied complex is the hetero-hexameric SEPT2/6/7 complex (19, 20). These septin complexes polymerize to form filaments, which are thought to be the primary biologically active form of septins (21).

In cells, septin filaments and F-actin co-localize at multiple structures (22, 23, 24, 25, 26, 27, 28, 29). Knock-down of septins disrupts acto-myosin stress fibers, suggesting a role for septins in maintaining the integrity of stress fibers (22, 30). However, the mechanism underlying this requirement for septins in stress fiber maintenance has remained unclear. *In vitro*, septin filaments directly bind microtubules (27) and SEPT9 directly binds and bundles both actin and microtubules (2, 12, 31). Septin complexes directly bind actin *in vitro* as well (32, 33). Both direct septin-actin binding and adaptor-protein mediated septin-actin interactions have been proposed (22, 32, 33, 34, 35). The molecular and structural details controlling septin-actin interactions have only begun to be revealed.

The Cdc42 effector proteins (Cdc42EPs), also known as Binder of Rho GTPase proteins (BORGs) (36, 37), have been implicated in regulating septin-actin interactions. Cdc42EPs bind to septins and Cdc42/TC10 and play important roles in cell motility, angiogenesis, glutamate clearance, and cancer progression (14, 36, 37, 38, 39, 40, 41, 42, 43, 44, 45). Altering the levels of Cdc42EPs changes septin and actin organization in cells in multiple contexts (14, 22, 36, 37). For example, Cdc42EP3 is overexpressed in cancer-associated fibroblasts (CAFs), where it plays an important role in CAF activation by promoting mechanotransduction *via* stress fiber and septin filament formation (22). In HHL60 cells, paclitaxel-induced proteasomal degradation of Cdc42EP3 and Cdc42EP5 results in the relocalization of septins from actin stress fibers to microtubules, concomitant with stress fiber disassembly (46). Both studies illustrate the *in vivo* significance of Cdc42EP3 in controlling actin and septin co-localization and stability, and how alteration of Cdc42EP3 levels has physiologically relevant consequences.

Cdc42EP3 is also upregulated in multiple cancers, including glioma, osteosarcoma, gastric cancer, and colorectal cancer (43, 47, 48, 49). In each of these cancers, knocking down Cdc42EP3 reduced cell viability, proliferation, impaired migration, increased apoptosis, and tumors in mice were smaller. While septin and actin distributions have not been directly examined in these cells, it is evident that Cdc42EP3 has strong physiological relevance in the context of cancers and cancer-related pathologies. An expanded understanding of its mechanisms of action could identify a new target for therapeutic development. Despite this growing importance, the mechanism by which Cdc42EP proteins, and especially Cdc42EP3, promote septin-actin interactions remains poorly understood.

The Cdc42EP family proteins possess multiple protein-protein interaction motifs, including a Cdc42- and Rac-Interactive Binding motif (CRIB) and the BORG Homology 3 (BH3) motif (36, 37). The CRIB motif binds to the Rho family GTPases Cdc42 and TC10 (36, 37). The binding of Cdc42 to Cdc42EPs is important for many of Cdc42EP’s cellular functions (37, 41, 44, 50, 51). The BH3 motif is a conserved element that likely binds at the interface of SEPT6 and SEPT7 (45). The consequences of BH3 binding to septins are poorly understood, but available evidence suggests that the interaction may stabilize septin filaments (22, 44, 52). The BORG-Homology region 1 (BH1) domain is conserved across Cdc42EPs, while the BORG-homology region 2 (BH2) domain is present in all family members except for Cdc42EP5. In addition, all Cdc42EP family members have a basic motif upstream of the CRIB, although these vary in size. The functions of the basic region, BH1 and BH2 domains are unknown (36, 37).

Here, using biochemical reconstitution, we investigated how purified Cdc42EP3 influences the interactions and effects of septins on actin. We find that Cdc42EP3 binds directly to actin monomers and filaments and that Cdc42EP3-bound septin filaments nucleate actin polymerization.

## Results

### Biochemical characterization of purified Cdc42EP3 and its interaction with septins

**Figure 1:**
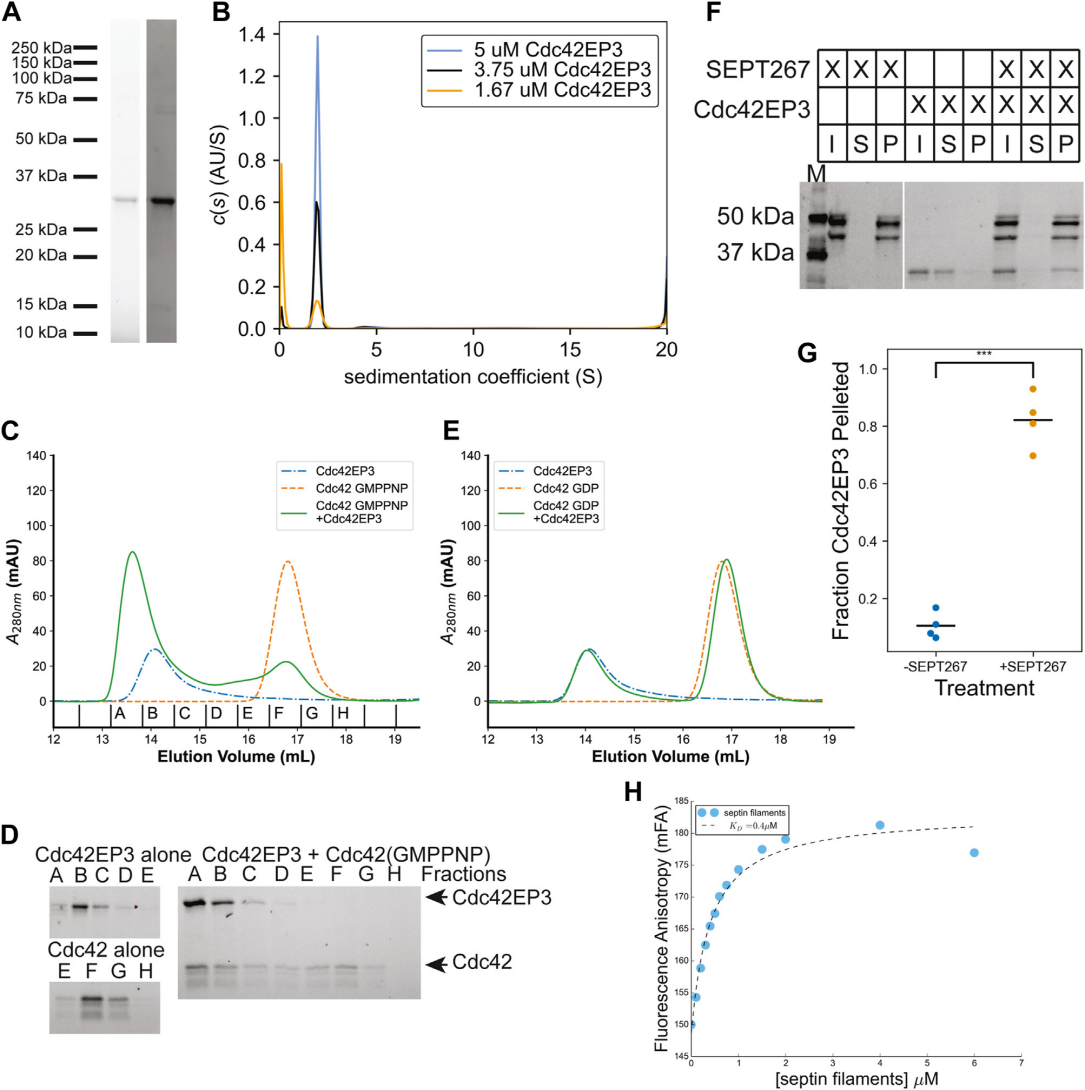
**Purification and biochemical characterization of Cdc42EP3**. *A*, purified Cdc42EP3 resolved using SDS-PAGE, imaged using “Stain-Free” imaging (*left*) and Coomassie Brilliant Blue (CBB) stain (*right*). *B*, *c*(*s*) distributions of Cdc42EP3 at three different concentrations measured using SV-AUC. *C*, gel filtration chromatograms of Cdc42EP3, GMPPNP-loaded Cdc42 or both proteins. *D*, SDS-PAGE Stain-Free imaged gel of gel filtration fractions from (*C*). Letters in gel correspond to the lettered fractions in (*C*). *E,* gel filtration chromatograms of Cdc42EP3, GDP-loaded Cdc42 or both proteins. *F*, co-pelleting of Cdc42EP3 with SEPT2/6/7 filaments. Samples were centrifuged at 21,000*g* for 20 min, supernatant fraction was removed and pelleted fractions were resuspended in high salt buffer. Input (I), Supernatant (S) and Pelleted (P) fractions were analyzed using SDS-PAGE and imaged using Stain-Free imaging. *G*, band intensity quantification and two-tailed *t* test of the fraction of Cdc42EP3 that pelleted alone compared to with septin filaments, shown as swarm plot. ∗∗∗ means *p* < 0.001. The line represents the mean. *H*, fluorescence anisotropy assay measuring septin filament binding to Cdc42EP3.

While the collection of cellular functions ascribed to Cdc42EPs is growing, the mechanism of Cdc42EP function is poorly understood. To explore these mechanisms *in vitro*, we purified recombinant Cdc42EP3 (Fig. 1*A*). Attempts to purify Cdc42EP3 using a previously described GST fusion (44, 45) yielded poorly behaved materials in our hands (not shown). Thus, we employed a novel strategy involving an MBP-fusion followed by cleavage and cation exchange, which improved purification considerably (Fig. S1, *A* and *B*). Our recombinant Cdc42EP3 was homogenous, as assessed using gel-filtration analysis (Fig. S1, *C* and *D*). Sedimentation-velocity analytical ultracentrifugation (SV-AUC) revealed a homogeneous population with a concentration-independent sedimentation coefficient, arguing against oligomerization (Figs. 1*B* and S1*E*). Integration of the *c*(*M*) distribution for each sample yields molecular weight estimates between 23 and 27 kDa, consistent with a monomer of Cdc42EP3 (Fig. S1*F*). The frictional ratio found in SV-AUC analysis was >1.50 (Fig. S1*F*), indicating a partially extended structure, consistent with the early elution on gel filtration (Fig. S1, *C* and *D*). We conclude that Cdc42EP3 is monomeric and that it is not compactly folded.

To assess the functionality of our recombinant Cdc42EP3, we tested its ability to bind septins and Cdc42. In gel-filtration assays, Cdc42EP3 bound to Cdc42 in its GTP-bound state but not in its GDP-bound state, which was expected (Fig. 1, *C*–*E*). To test binding to septins, we used a sedimentation assay with septin filaments assembled from the most widely studied septin oligomers, composed of SEPT2, SEPT6, and SEPT7 (called ‘SEPT2/6/7’ hereafter). Cdc42EP3 co-sedimented with septin filaments, indicating a direct interaction (Fig. 1, *F* and *G*). Therefore, our purified Cdc42EP3 is functional with respect to Cdc42 and septin binding.

Cdc42EP3 labeled with tetramethylrhodamine (TMR) at C211 (Fig. S1*G*) was used to quantify binding to septin filaments by measuring changes in its fluorescence anisotropy (F.A.). Free Cdc42EP3 F.A. values should be low compared to the filament-bound state. As SEPT2/6/7 filaments were titrated into the reactions, F.A. values increased and eventually saturated (Fig. 1*G*). TMR-fluorescence intensity was not substantially changed during this titration (not shown). From these data, we determined the affinity of Cdc42EP3 for SEPT2/6/7 filaments to be 0.4 μM ± 0.1 μM (Fig. 1*G*, dashed line). These provide the first quantitative assessment of a Cdc42EP affinity for septins.

### Cdc42EP3 directly binds to actin

**Figure 2:**
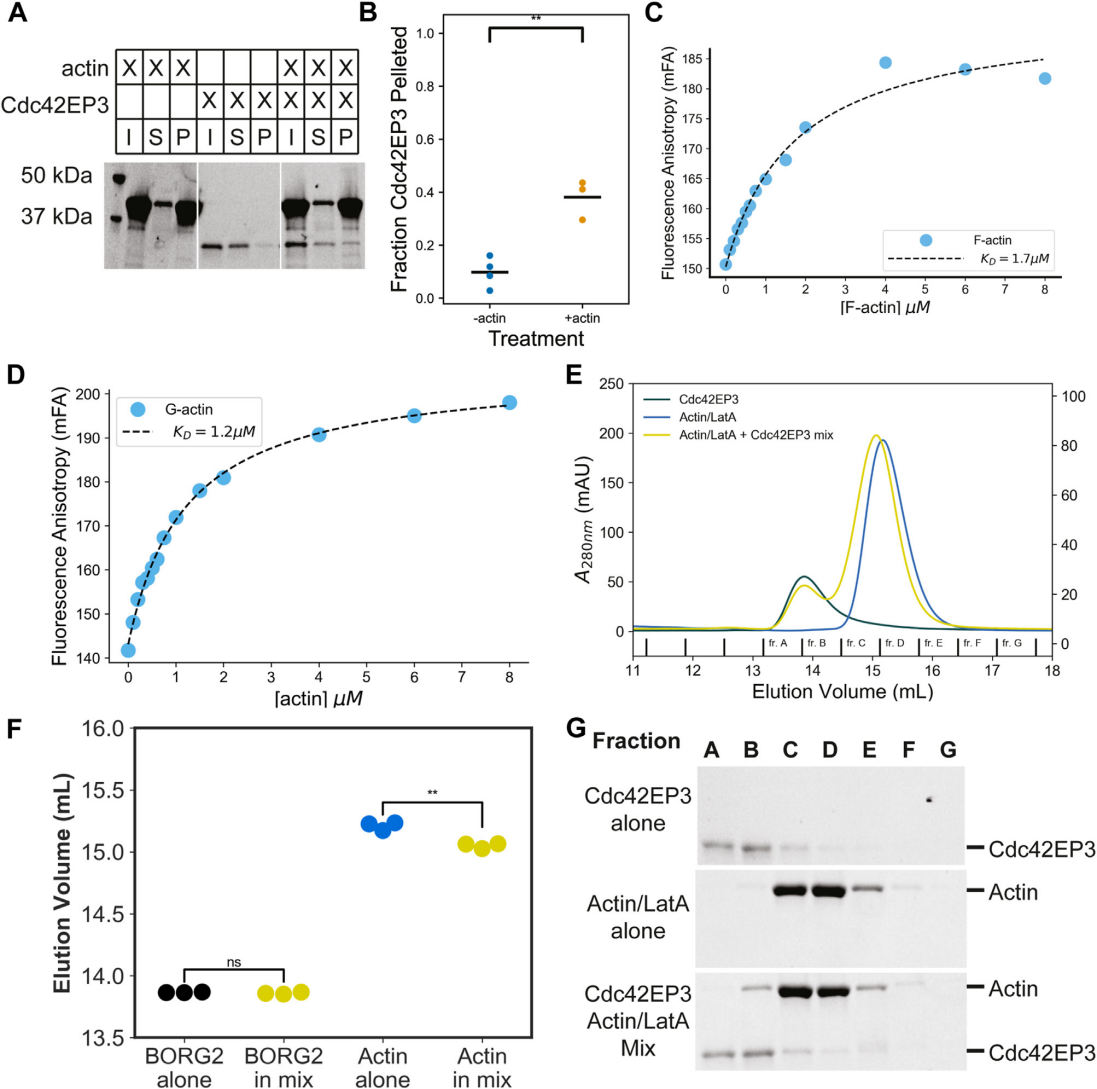
**Cdc42EP3 binds actin**. *A*, centrifugation assay with actin filaments, Cdc42EP3, or both. Samples were centrifuged at 100,000*g* for 1 h and separated into supernatant and pelleted fractions. *B*, quantification of pelleted band intensity and two-tailed *t* test of the fraction of Cdc42EP3 that pelleted alone *versus* in the presence of actin filaments. ∗∗ means *p <* 0.01. Data are shown as a swarm plot, the line represents the mean. *C*, fluorescence anisotropy assay measuring actin filament (F-actin) binding to Cdc42EP3. Cdc42EP3 was maintained at a fixed 20 nM concentration while an increasing amount of actin filaments were titrated. *D*, Fluorescence anisotropy assay measuring Latrunculin A bound actin monomer binding to Cdc42EP3. Cdc42EP3 was maintained at a fixed 20 nM concentration while increasing amount of actin monomer was titrated in with 20 μM Latrunculin A. *E*, chromatogram of analytical gel filtration of Cdc42EP3, monomeric actin or a sample containing both, representative example from n = 3 repeats. *F*, two-tailed *t* test of elution volumes in (*E*). ∗∗ means *p* < 0.01. Data are shown as swarm plot and the line represents the mean. *G*, SDS-PAGE Stain-Free imaging of fractions corresponding to the gel filtration experiment in (*E*).

An interaction between Cdc42EP3 and F-actin has been proposed (22), and therefore we directly tested this idea using purified Cdc42EP3. In actin filament spin-down assays, Cdc42EP3 pelleting increased in the presence of actin filaments (Fig. 2, *A* and *B*) consistent with F-actin binding. F.A. assays confirmed that Cdc42EP3 binds to actin filaments, with an affinity of 1.7 μM ± 0.4 μM (Fig. 2*C*). Cdc42EP3 also associated with latrunculin-bound actin monomers with an affinity of 1.2 μM ± 0.1 μM (Fig. 2*D*). Consistent with the strength of this interaction, in gel filtration experiments we found that Cdc42EP3 and actin monomers did not elute as a stable complex; however, the mixture reproducibly shifted the actin elution to an earlier fraction (Fig. 2, *E*–*G*). Thus, Cdc42EP3 binds to both F-actin and G-actin with similar affinities.

**Figure 3:**
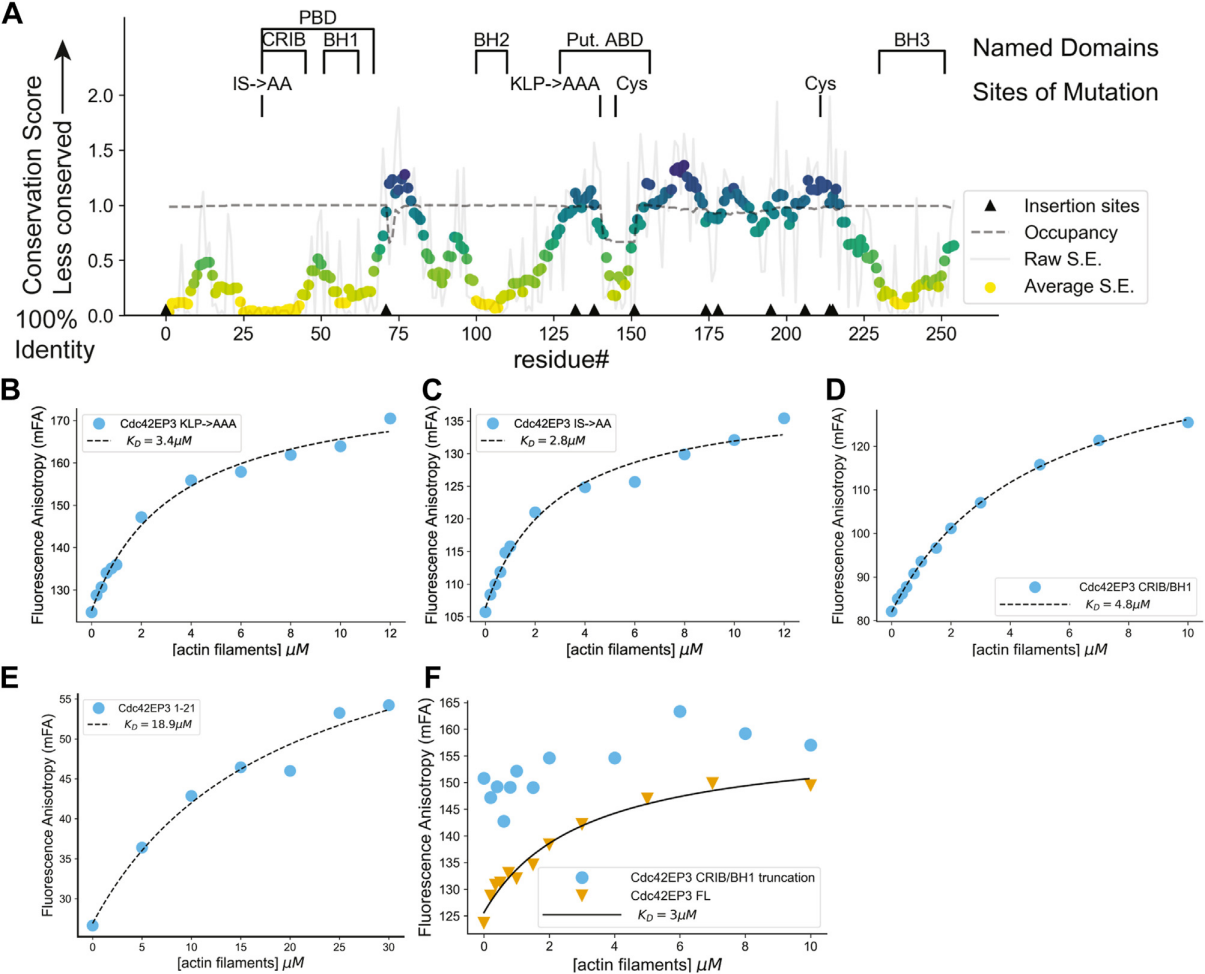
**The N-terminal CRIB/BH1 region of Cdc42EP3 binds actin**. *A*, conservation scores for residues in human Cdc42EP3, where conservation score is the Shannon Entropy. Values range from 0 to 3 with 0 being fully conserved (100% identity) and 3 the least conserved. The locations of named regions, endogenous cysteines and targeted mutations are indicated. Occupancy indicates the fraction of sequences with a residue present in a gapped alignment (1.0 means all sequences have a residue). *Arrows* indicate sites of insertions in non-human sequences. Fluorescence anisotropy assay measuring binding to actin filaments of TMR labeled (*B*) 20 nM KLP-AAA Cdc42EP3 mutant, (*C*) 20 nM IS-AA Cdc42EP3 mutant, (*D*) 20 nM isolated Cdc42EP3 CRIB/BH1 domains, or (*E*) 5 nM Cdd42EP3 (1-21) was maintained at fixed concentration while increasing amount of actin filaments were titrated in. TMR label introduced at C211 (*B* and *C*) or at the N-terminus (*D* and *E*). *F*, fluorescence anisotropy overlay of FL/WT Cdc42EP3 binding to actin filaments *versus* Cdc42EP3 binding to actin filaments when the CRIB and BH1 regions have been removed. Truncation did not produce a reliable F.A. change and was not fit. When fit, *K*_*D*_ is indicated in the legend.

A prior study identified two sites that when mutated alter actin binding in cellular assays, one in the CRIB region and the other in a region of unknown function (residues 127–156, see Fig. 3*A*) (22). We produced new versions of the Cdc42EP3-TMR F.A. probe, each with one of these mutation clusters (see Fig. 3*A* for location of mutations) (22). In F.A. assays with F-actin, both mutant probes bound to F-actin with a similar affinity to wild-type Cdc42EP3 (Fig. 3, *B* and *C*), in contrast to the prior findings. Therefore, we turned to truncations to better delineate the actin-binding region of Cdc42EP3.

To inform our truncation design, we scored sequence conservation among vertebrate Cdc42EP3 orthologs using Shannon entropy (Fig. 3*A*) (36, 37, 53, 54). A notable feature in our Cdc42EP3 alignment is that the CRIB and BH1 regions are highly conserved and contiguous. We propose that together these comprise a single unit, which is supported by the identification of the CRIB and BH1 regions as a single unit by the NCBI Conserved Domain Database (55). Moreover, many CRIB motif-containing proteins have regions following the CRIB that improve affinity for Cdc42 (56, 57, 58). Therefore, we focused on constructs including or lacking the CRIB-BH1 region.

To identify which region of Cdc42EP3 is needed for F-actin binding, we produced truncated Cdc42EP3 lacking the conserved-N-terminal region (including the CRIB-BH1) or including only this region. The isolated N-terminus of Cdc42EP3 bound to F-actin with only two-fold reduced affinity compared to full-length Cdc42EP3 (Fig. 3*D*). Further truncating this to the first 21 amino acids yielded a material that is still bound to F-actin, albeit with a further 3-4-fold weakened affinity (Fig. 3*E*). Conversely, a Cdc42EP3 construct *lacking* the N-terminus through the CRIB/BH1 region did not bind F-actin (Fig. 3*F*). From these results, we conclude that Cdc42EP3 binds to F-actin using an unrecognized N-terminal region.

### SEPT2/6/7 polymers directly associate with actin filaments

**Figure 4:**
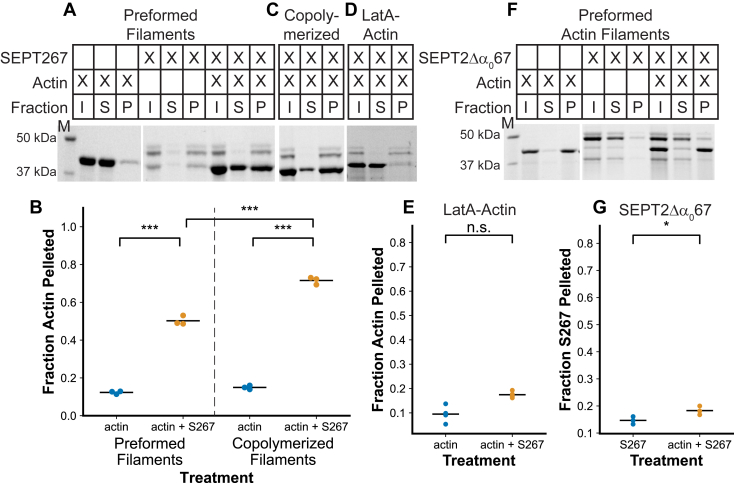
**Septin polymers directly bind actin filaments**. *A*, septins and actin were separately polymerized, mixed, and incubated on ice and centrifuged at 21,000*g*. *B*, quantification of band intensities of pelleted actin in the presence or absence of septins. *C*, septins and actin were mixed and co-polymerized while undergoing dialysis overnight, then centrifuged at 21,000*g* the following day. *D*, Pre-formed septin filaments were mixed and incubated with Latrunculin A bound actin monomers and spun down at 21,000*g*. *E*, quantification of band intensities of pelleted actin from *D* in the presence or absence of septins. *F*, pre-formed actin filaments were incubated with a non-polymerizable SEPT2/6/7 hexamer and then centrifuged at 100,000*g*. *G*, quantification of band intensities of pelleted septin hexamer in the presence or absence of actin filaments. For *A*, *C*, *E*, and *G* Input (I), Supernatant (S), and Pelleted (P) fractions were analyzed using SDS-PAGE and imaged using Stain-Free imaging. For *B*, *E*, and *G*, fractions of actin or septin pelleted are shown as swarm plots (*horizontal lines* show the mean). *B*, a one-way ANOVA followed by Tukey-HSD posthoc was performed with n = 3 for each group. ∗∗∗ indicates *p* < 0.001. *E* and *G*, A two-tailed *t* test was performed with n = 3 for each group. ∗ indicates *p* < 0.05 and ∗∗∗ indicate *p* < 0.001.

Although initial studies led to conflicting evidence for whether SEPT2/6/7 filaments interact with actin filaments, recent evidence has more strongly supported the notion that F-actin and septin filaments directly associate (28, 32, 33, 59). When we separately polymerized actin and SEPT2/6/7 septins and then mixed them, some actin co-pelleted with septins at low speed (Fig. 4, *A* and *B*). When SEPT2/6/7 and actin were co-polymerized, actin filaments pelleted with the septin filaments more efficiently than when they were separately polymerized (Fig. 4, *B* and *C*). G-actin failed to co-sediment with SEPT2/6/7 septin filaments (Fig. 4, *D* and *E*). Similarly, F-actin only slightly increased the sedimentation of a nonpolymerizable septin hexamer missing part of the SEPT2-SEPT2 interface (Fig. 4, *F* and *G*). Therefore, actin and septins interact specifically when both are in filamentous form, and their interaction is enhanced by co-assembly.

**Figure 5:**
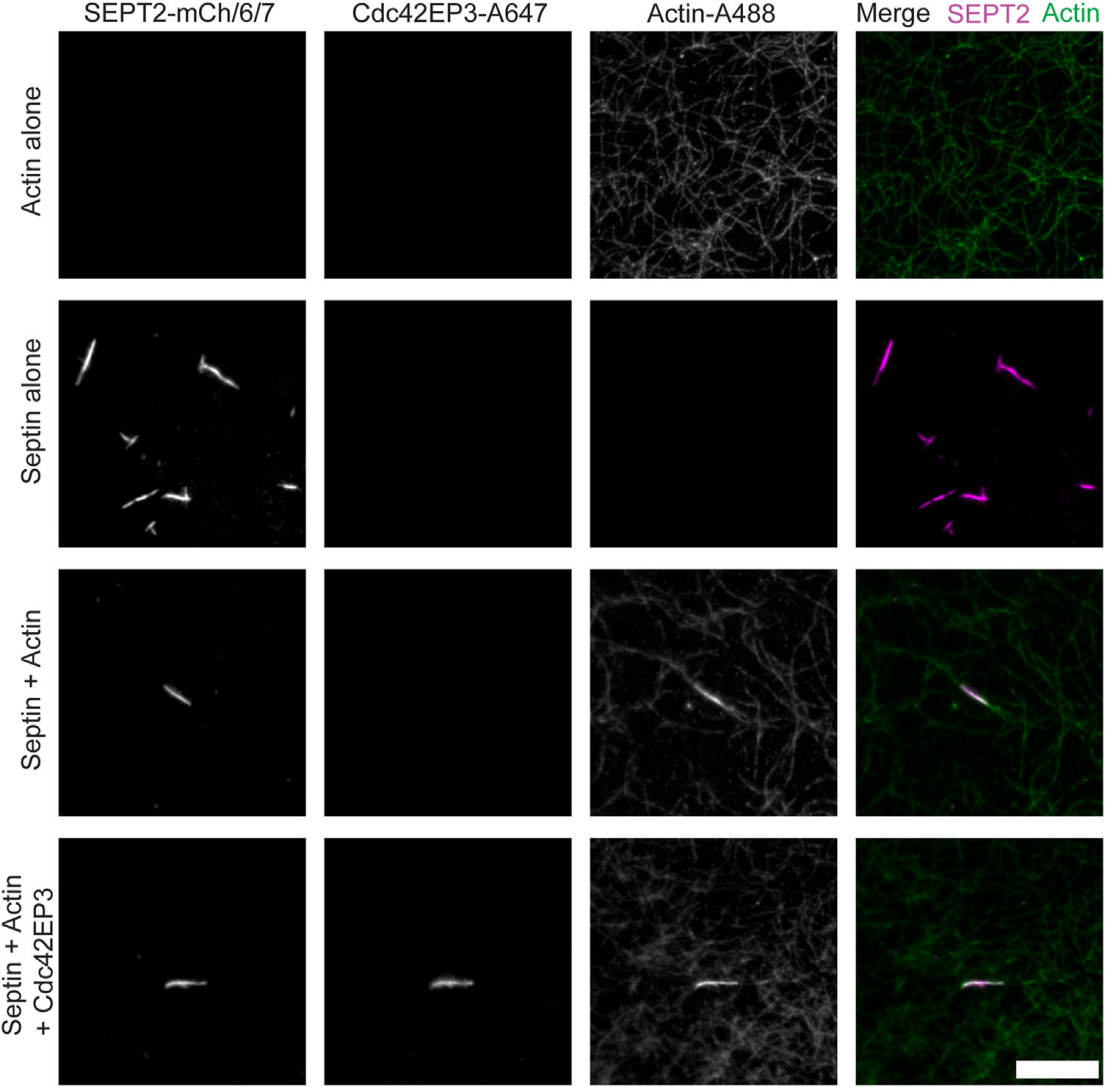
**Co-polymerization and alignment of septins and actin *in vitro***. Representative fields of view for TIRF imaging of *in vitro* reconstituted mCherry labeled SEPT2/6/7 filaments, Alexa 488 labeled actin filaments, and Alexa 647 labeled Cdc42EP3 under hydrated conditions. Horizontal groups represent different channels and actin-septin merge composites for each sample. Reactions contain, as indicated, 2 μM actin (10% Alexa488-labeled, 2% biotinylated), 1 μM SEPT2-mCherry/SEPT6/SEPT7-StrepII tag filaments, and/or 700 nM Alexa647-Cdc42EP3. Fields of view were imaged for Alexa488-actin (*green*), for SEPT2-mCherry (*magenta*) or for Alexa647-Cdc42EP3. On the right, two-color merge with SEPT2 colored *magenta* and actin *colored green*. All panels are at the same scale and the scale bar at the *lower right* is 10 μm.

To assess the organization of actin and septin filaments, we visualized septin-actin structures after incubation for at least 1 hour. Separately, SEPT2/6/7 and actin each formed filaments (Fig. 5). When co-polymerized with actin, SEPT2/6/7 formed filaments that are grossly similar to those observed for SEPT2/6/7 alone, but now they aligned with F-actin. Further, the intensity of F-actin in regions of overlap was 7.1 ± 0.5 (S.E.M) times higher than actin filaments in the absence of SEPT2/6/7 (Figs. 5 and S2*A*). These observations are in good agreement with our sedimentation data and support the view that the septin filaments bundle F-actin.

We next asked whether Cdc42EP3 recruits actin to the septin filaments. Adding Cdc42EP3 to septin-actin filament co-pelleting assays did not increase actin co-pelleting with preformed SEPT2/6/7 filaments (Fig. S2*A*). In these total internal reflection fluorescence (TIRF) microscopy imaging experiments, Cdc42EP3 had a minimal effect on the appearance of the septin and actin filaments (Fig. 5), and Cdc42EP3 did not significantly change the intensity of F-actin bundles at regions of septin overlap (Figs. 5 and S2*B*). Cdc42EP3 localized to the septin-F-actin overlap region, but not to the regions of F-actin devoid of septins. These observations likely reflect the higher affinity of Cdc42EP3 for septins and the concentration of Cdc42EP3 being limiting, a design that mimics the best existing concentration estimates available (60). We conclude that while Cdc42EP3 decorates septin-F-actin co-polymer regions, it does not substantially impact their organization.

### Cdc42EP3 accelerates actin polymerization in the presence of septins

Septins and Cdc42EPs co-localize with cellular actin structures, most notably stress fibers (22, 25, 29, 30). While not part of the canonical stress-fiber assembly process (61, 62, 63), these proteins are important for stress-fiber integrity, as depleting septins or Cdc42EP3 disrupt stress fibers in multiple contexts (22, 46). To better understand the mechanistic basis for these *in vivo* effects, we tested whether Cdc42EP3 and/or septins might stabilize actin filaments against their net disassembly induced by the actin monomer-sequestering agent Latrunculin A. F-actin polymerized with septins and/or Cdc42EP3 was equally susceptible to depolymerization by Latrunculin A, as evidenced by indistinguishable depolymerization in pyrene actin assays (Fig. S3*A*). Therefore, we conclude that septins and Cdc42EP3 do not stabilize F-actin structures against depolymerization.

**Figure 6:**
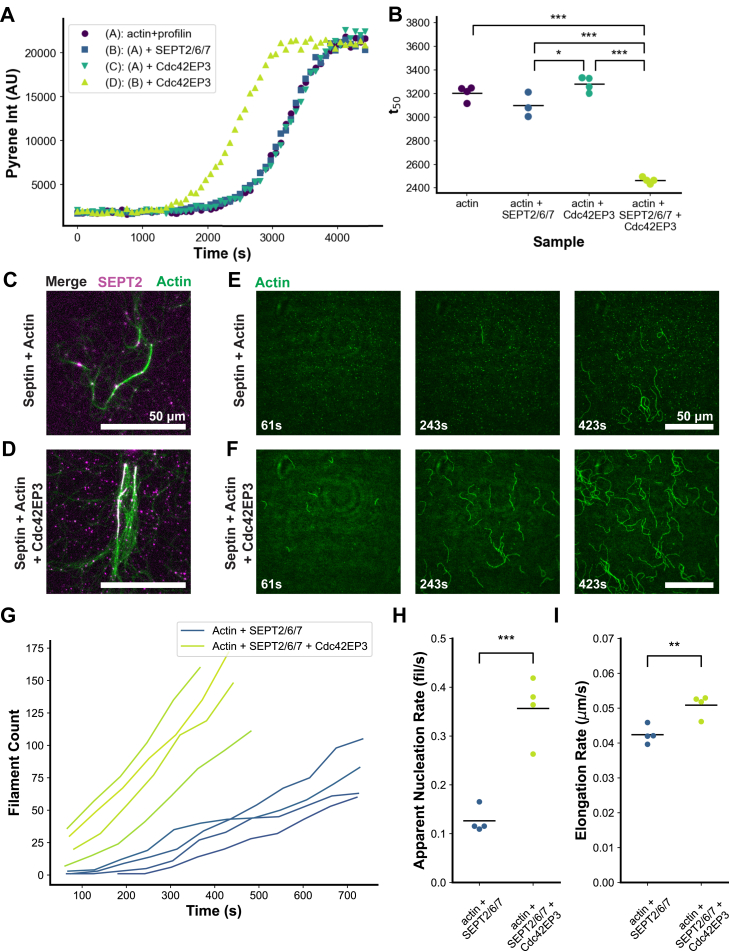
**Cdc42EP3-septin complexes promote actin polymerization**. *A*, 2 μM actin monomers (10% pyrene labeled) and 10 μM profilin were mixed with polymerization buffer and 500 nM Cdc42EP3 and/or 500 nM SEPT2/6/7 filaments, and actin polymerization was monitored by the increase in pyrene fluorescence intensity over time until polymerization was complete. Only every 15th data point is shown. *B*, the time to reach 50% complete polymerization (*t*_*50*_) was quantified for each sample. ∗ is *p* < 0.05 and ∗∗∗ is *p* < 0.001, using one-way ANOVA followed by Tukey *post hoc* test. Data shown as swarm plot, with the line representing the mean. *C* and *D*, 2 μM actin (10% Alexa488 labeled, 0.25% biotinylated) with 1 μM SEPT2/6/7 filaments (10% mCherry-SEPT2 labeled) form actin filament bundles comparable to those seen in Figure 5 in the absence (*C*) and presence (*D*) of 500 nM Alexa647-Cdc42EP3. *E* and *F*, images from early time points in TIRF actin assembly assays, showing actin filament assembly in the presence of septins or Cdc42EP3, as indicated at *left*. Elapsed time from mixing of septin and actin is shown at the *lower left* of each frame. *C–F*, scale bars are 50 μm. *G*, Filament counts over time for four different fields of view for the indicated samples. *H*, apparent nucleation rates (filament appearance within TIRF field in field of view). *I*, filament elongation rates from the same reactions. Each point is the average of at least 20 filaments in a field of view. *H* and *I*, individual data points are shown as swarm plots, with means shown as *horizontal lines*. Two-sample, two-tailed *t* test was used to determine significance, with ∗∗ as *p* < 0.01 and ∗∗∗ as *p* < 0.001.

Next, we used pyrene assays to ask whether Cdc42EP3 and septins alter the kinetics of actin assembly. Initial experiments adding septin and Cdc42EP3 to actin revealed only a subtle change in polymerization rate (Fig. S3*B*). However, adding septin and Cdc42EP3 to actin in the presence of profilin accelerated actin polymerization (Fig. 6, *A* and *B*). Importantly, this effect was not observed with either septins or Cdc42EP3 alone and therefore suggests that septin-Cdc42EP3 complexes modestly promote actin assembly.

To directly visualize actin assembly from septin filaments with Cdc42EP3, we used 3-color TIRF video microscopy. Labeled SEPT2/6/7 was first assembled into filaments by dialysis, in the presence or absence of Cdc42EP3, and then mixed with actin monomers (10% AlexaFluor488-labeled and 0.25% biotin-labeled) and 10 μM profilin under conditions that supported assembly of both actin and septin filaments. At longer time points similar septin-actin bundles were observed to those in Figure 5, with or without Cdc42EP3 (Fig. 6, *C* and *D*). Focusing on earlier time points, individual actin filaments were observed falling into the TIRF plane and attaching (Fig. 6, *E* and *F*). By tracking their appearance over time, filament counts should approximate the nucleation rate. Note that the exact nucleation rate cannot be determined from these experiments because filaments can both nucleate in the TIRF plane or above it and then fall into the plane (there is evidence of both happening). Tracking filament counts over time, we found that the addition of SEPT2/6/7 filaments with Cdc42EP3 resulted in a more rapid accumulation of actin filaments than SEPT2/6/7 alone (Fig. 6, *G* and *H*). This supports the hypothesis that SEPT2/6/7-Cdc42EP3 complexes modestly stimulate actin filament nucleation. On the other hand, SEPT2/6/7 and Cdc42EP3 had minimal effects on the elongation rate of actin filaments (Fig. 6*I*), again supporting our conclusion that SEPT2/6/7 with bound Cdc42EP3 promotes actin filament nucleation from septins.

## Discussion

A previous description of Cdc42EP3 in CAF cells identified a ‘putative actin-binding site’ that was unique to Cdc42EP3 (22). Here, we have confirmed that Cdc42EP3 binds actin, measured the affinities, and narrowed down the region of Cdc42EP3 that mediates these interactions. We found that Cdc42EP3 binds to both actin filaments and actin monomers (Fig. 2). In contrast to the previous study (22), our data places the actin-binding site at the N-terminus (Fig. 3). This includes the CRIB region and a basic region found across the Cdc42EP family, suggesting that other Cdc42EP family members may also bind actin. Notably, Cdc42EP5 (also known as BORG3) has a similar role in melanoma metastasis as does Cdc42EP3 in promoting cancer-associated fibroblast activation (22, 42). Moreover, both Cdc42EP3 and Cdc42EP5 are involved in maintaining septin filaments in association with and stabilizing stress fibers (46). Conversely, Cdc42EP1 depletion was found to promote radial stress fiber formation (23). Cdc42EP1 has only a 3 amino acid long N-terminal ‘basic region’ indicating substantial differences in the region we have identified as responsible for actin binding (Fig. 3). These new insights open future exploration of the actin-binding roles across the Cdc42EP family.

We have found that septin (SEPT2/6/7) filaments bind to actin filaments, but not actin monomers (Fig. 4, *A*–*E*), and that actin filaments do not efficiently associate with unpolymerized septin oligomers (Fig. 4, *F* and *G*). This largely resolves the existing and conflicting reports with respect to actin and septins interacting (32, 33). Pre-formed actin filaments and septin filaments do not interact as well as co-polymerized septins and actin (Fig. 5, *A*–*C*), but they do interact. In addition to the order of filament assembly making a difference, there may be a time and concentration-dependent aspect to these interactions, which may also partly explain the existing conflicting reports. The mechanism behind the improved interaction of co-polymerized filaments could be as simple as slow diffusion by filament assemblies preventing contact between filament types. A diffusion-limited interaction is consistent with the previous observation of actin filament decoration by septin complexes that have not polymerized (32, 59).

**Figure 7:**
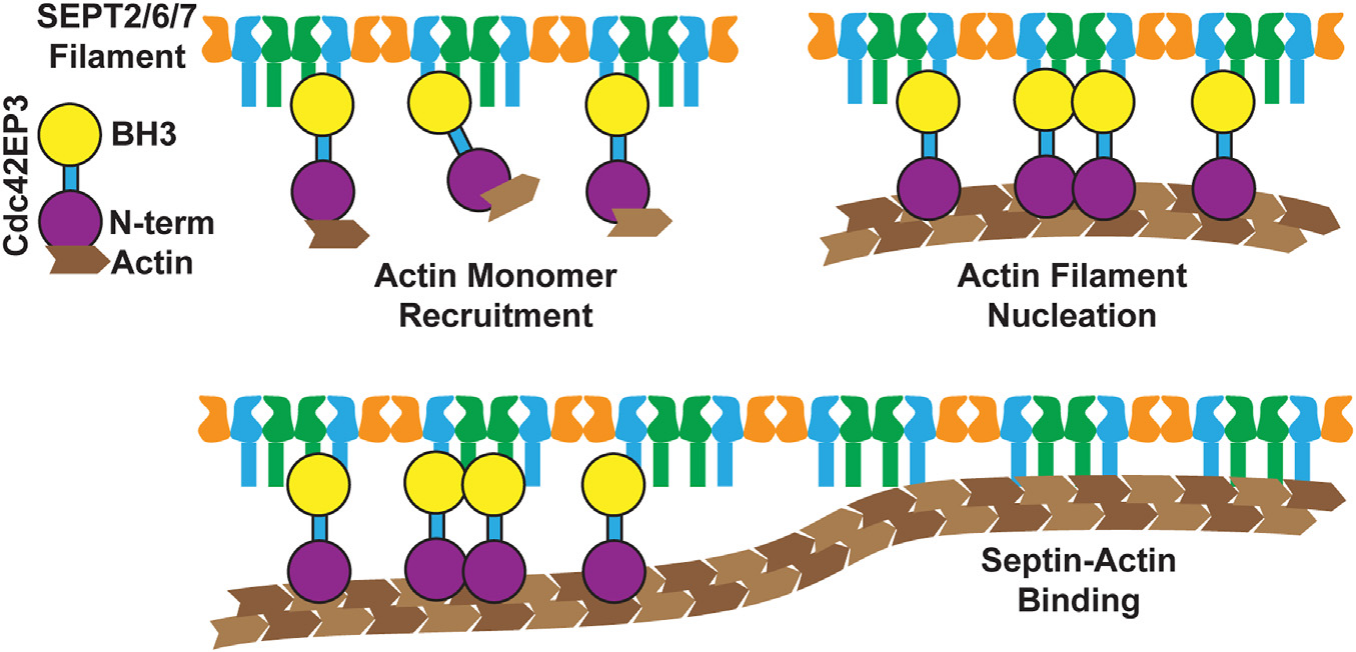
**Model for Cdc42EP3 promotion of actin assembly on septin scaffolds**. Cartoon depiction of the mechanism by which Cdc42EP3 promotes actin assembly.

What role does the Cdc42EP3-actin interaction serve? We initially hypothesized that Cdc42EP3 may stabilize the interaction between septin and actin filaments (22). Instead, we found that septin-actin interactions were stable in the absence of Cdc42EP3, suggesting a kinetic role for Cdc42EP3. Cdc42EP3 binds actin monomers and actin filaments (Fig. 2), while septin filaments (SEPT2/6/7) bind actin filaments, but not monomers (Fig. 4). A possible mechanism promoting actin polymerization is that Cdc42EP3 binds actin monomers when arrayed on septins, increasing the local actin-monomer concentration and promoting filament nucleation (Fig. 7). These actin filaments then immediately bind to the septin filaments due to their proximity, forming stable septin-actin (‘septactin’) bundles. This role of Cdc42EP3 could explain why septin filaments alone minimally alter actin polymerization rates. Our experiments have focused on SEPT2/6/7 filaments, as SEPT9 has actin-binding activity on its own (2, 31). While the synergy or redundancy of Cdc42EP3 and SEPT9 remains to be determined, Cdc42EP5 has similar roles as SEPT9 in melanoma cells, where both Cdc42EP5 and SEPT9 are required for actomyosin-driven cell migration (42).

How do our results impact our mechanistic understanding of the *in vivo* role of Cdc42EP3 in stabilizing stress fibers? In the prevailing model of stress fiber assembly, antiparallel-actin bundles are held together (crosslinked) by α-actinin and assembled with non-muscle-myosin II rods to form the repeating contractile structure (61, 62, 63). While several studies have shown that septin expression impacts stress fiber stability (2, 22, 46, 64) neither septins nor Cdc42EP proteins are conventionally thought of as essential for forming stress fibers. We speculate that septin-actin bundles might replace the actin-α-actinin bundles in select stress fiber types. Due to the bipolar symmetry of hexameric and octameric septin rods (65), associated actin filaments likely have an antiparallel organization, similar to α-actinin bundles. The assembly of the α-actinin-stabilized actin bundles has a major advantage over septin filament clusters, as α-actinin should diffuse more quickly than septin filament structures. Thus, in the context of septin filament bundled actin, it may be more kinetically favorable to assemble actin filaments from actin monomers directly on the septin filament surface through Cdc42EP3-dependent nucleation instead of capturing preassembled actin filaments from the cytoplasm. Alternatively, Cdc42EP3 may also selectively stabilize the septin filaments associated with actin (22). Consistent with our ideas above, SEPT2 knockdown in MDCK cells results in disorganized focal adhesions and actin cortex that can be rescued by overexpression of α-actinin (2).

Our biochemical experiments also provide new mechanistic insights into the *in vivo* function of Cdc42EP3 in maintaining septin-actin structures. In CAFs, depletion of Cdc42EP3 results in the loss of septin-associated acto-myosin stress fibers, while overexpression Cdc42EP3 in normal fibroblasts triggers septin-associated stress fiber formation (22). Our *in vitro* findings provide a mechanism that could explain these effects, wherein Cdc42EP3 arrayed on septin filaments promotes actin filament assembly, initiating a new septin-actin bundle or enabling their persistence. The actin filaments in stress fibers undergo steady, dynamic turnover (66), implying that locally accelerating or decelerating actin assembly may result in enhanced or weakened stress fiber stability. This idea parallels the role of actin filament nucleation by APC in promoting focal adhesion stability and maturation (67, 68). These bundles could provide a persistent source of new actin filaments to replace those dynamically lost from the stress fibers. Notably, in HHL16 cells, Cdc42EP3 and Cdc42EP5 both stabilize septin-associated stress fibers (46). Upon paclitaxel treatment, Cdc42EP3 is lost from the septin-associated stress fibers prior to septin re-localization and stress-fiber disassembly (46). One interpretation of these data is that once the septin-actin structure is formed, Cdc42EP3 can be removed, leaving behind a temporary but non-self-renewing septin-actin structure. This view is consistent with stress-fiber stabilization roles proposed for Cdc42EP3 and Cdc42EP5 (22, 42) and with our finding that SEPT2/6/7-Cdc42EP3 assemblies promote actin filament assembly. Future cell biological studies focusing on the rates of septin and Cdc42EP protein turnover in stress fibers could provide insight into this mechanism.

## Limitations of the study

Several key limitations of the study are worth pointing out. First, our study is biochemical in nature and provides mechanistic context for the growing literature on cell-based studies examining Cdc42EP3 and other Cdc42EP proteins (see Introduction and Discussion sections). Second, throughout this study, we have used the SEPT2/6/7 hexamer as our model septin. Our current understanding of cellular mammalian septins is that they predominantly exist as octamers including a SEPT3 group septin, often SEPT9. This choice of model septin was made for two reasons (a) SEPT9 also binds to actin, which would substantially complicate the interpretation and (b) Cdc42EP family members had been described to bind in a fashion involving SEPT6/7, which is present in both the SEPT2/6/7 hexamers and octamers with SEPT9. Therefore, this represents the simplest reconstitution of septin-Cdc42EP3 interaction and the least prone to confounding effects. The role of Cdc42EP3 in the context of SEPT9 will need to be explored in the future. Third, our understanding of the assembly mechanism and assembly control for mammalian septins is still in its infancy. While our observations of the direct binding of actin monomers by Cdc42EP3 supports Cdc42EP3 dependent nucleation of actin filaments on septins, it is possible that some of the impact of Cdc42EP3 comes from the stabilization of septin assemblies. Finally, while our observation of actin filament nucleation by Cdc42EP3 on SEPT2/6/7 scaffolds is exciting, we did not directly observe actin filament nucleation on large, immobilized, septin structures (not shown, but related to examples in Figure 4, *C* and *D*). Instead, at early time points nucleated actin filament largely fell into the TIRF field without septins or with only small SEPT2/6/7 structures associated. Larger septin-actin bundles appeared later in the time course and it is not clear what kinetic events lead to the larger bundles. Thus, the model proposed in Figure 7 applies to the context shown—individual septin and actin filaments—but larger septin-actin bundles probably arise through additional kinetic steps.

## Experimental procedures

### Cdc42EP3 purification

A codon-optimized coding sequence for Cdc42EP3 was ordered as a synthetic dsDNA fragment (gBlock) from IDT DNA. The synthetic portion included unique NdeI and BamHI restriction sites and a coding region for a C-terminal His_6_ tag. This was inserted into a modified pMAL2C vector (New England Biolabs), which had a TEV protease cleavable MBP fusion and a modified multiple cloning site (vector was a gift from Michael K. Rosen, University of Texas Southwestern Medical Center). Cloning was accomplished *via* restriction digestion targeting introduced NdeI and BamHI restriction sites, followed by ligation into the target vector with T4 DNA ligase. The resulting vector was validated by Sanger sequencing.

The vector for expressing MBP-Cdc42EP3-His6 was transformed into BL21(DE3)-T1R *E. coli* bacteria, grown in Luria Bertani media at 37 °C to an OD_600_ of ∼0.8 and induced with 1 mM IPTG for 3 hours at 37 °C. Cultures were resuspended in Lysis Buffer (20 mM Tris/HCl (pH 8 at 20 °C), 200 mM NaCl, 1 mM EDTA, 1 mM DTT, and 1 mM PMSF) and snap frozen in liquid nitrogen. Cells were lysed by extrusion (Emulsiflex C5, Avestin Inc), and clarified by centrifugation at 17,000 RPM in a JA-20 rotor at 4 °C for 40 min. Clarified lysates were applied to Amylose High-flow beads (New England Biolabs) and washed extensively with AmyWB (20 mM Tris 8, 200 mM NaCl, 1 mM EDTA, 1 mM DTT, and eluted with AmyEB (20 mM Tris 8, 200 mM NaCl, 1 mM DTT, 1 mM EDTA, 30 mM Maltose). The highest concentration amylose elutions were pooled and TEV protease was added to cleave off MBP overnight at 4 °C. The sample was diluted 1:5 with Buffer A (20 mM HEPES pH 7.0, 0.5 mM EDTA, 1 mM DTT) and applied to a Mono S 5/50 Gl column, from which Cdc42EP3 eluted at approximately 22% Buffer B (Buffer A with 1 M NaCl added). Fractions were assessed by SDS-PAGE, pooled, supplemented with glycerol (20% w/v) flash frozen, and stored in −80 °C.

### Cdc42EP3 TMR labeling

Using site-specific PCR mutagenesis, single-cysteine Cdc42EP3 constructs were prepared (See Table S1). Cdc42EP3 proteins were purified as described above but with DTT omitted from the MonoS buffers. TMR labeling was accomplished through the addition of TMR-5-maleimide (T6027, Invitrogen/Thermo Fisher Scientific) in dry DMSO to the MonoS pool. Labeling proceeded for 1 h at 22 °C and then was quenched by the addition of 2 mM DTT. To remove excess dye, the quenched labeling reaction was applied to a Superdex 200pg 10/300 GL gel filtration column equilibrated in 150 mM NaCl, 10 mM HEPES/NaOH (pH 7 at 20 °C), 1 mM DTT. Preparation of Alexa647 labeled Cdc42EP3 used the same procedure, substituting Alexa647-maleimide for TMR-maleimide in the labeling of C145A Cdc42EP3 (unique cysteine is C211). Labeled Cdc42EP3 was quantified using absorbance of the fluorophore dyes, using extinction coefficients reported by the manufacturer, and stored as above.

### Purification and labeling of Cdc42EP3 truncations

Cdc42EP3 truncations lacking elements of the C-terminus or N-terminus were PCR amplified and inserted into the modified pMALC2 vector for purification (See Table S1). Truncations were expressed using the same protocol as full-length materials. The purification scheme for the C-terminal truncations is the same as the wild type (see above) while the N-truncations were purified by Amylose HF (NEB) followed by Ni Sepharose Fast Flow 6 beads (GE) and Superdex 200pg 26/600 size exclusion chromatography. TMR labeling and storage were as described above for the WT.

### Actin purification and labeling

Actin was purified from rabbit muscle according to the Spudich method (69, 70). Briefly, 5 g of rabbit muscle acetone powder (Pel-Freeze Biologicals) was twice extracted with Buffer G (2 mM Tris-HCl pH 8, 200 μM ATP, 0.5 mM DTT, 0.1 mM CaCl2, 1 mM sodium azide). The extract was then polymerized by the addition of magnesium chloride and potassium chloride, followed by depolymerization and size exclusion chromatography (Superdex 26/600 200pg column). Pyrene actin was prepared from actin depolymerized in Buffer G lacking DTT, which was polymerized by the addition of 50 mM KCl and 2 mM MgCl_2_, and then labeled with a ten-fold molar excess of N-(1-pyrene) iodoacetamide for 16 h at 4 °C. Oregon Green-labeled actin was prepared by dialysis against 50 mM KCl, 2 mM MgCl_2_, 10 μM ATP, and 2 mM HEPES/NaOH (pH 7.6 at 20 °C) to minimize amine content and to polymerize actin. Oregon Green 488 succinimidyl ester (Life Technologies) was added to 200 μM and allowed to react overnight at 4 °C. Labeled filaments were then collected, depolymerized, and purified by size-exclusion chromatography. Actins were stored in Buffer G in continuous dialysis.

### Purification of SEPT2/6/7 complexes

#### SEPT2/6/7 WT

A pCDF-derived plasmid expressing SEPT6 and SEPT7 and a pET15-derived plasmid expressing His6-SEPT2 were gifts from Elias Spiliotis. These two plasmids were co-transformed into BL21(DE3)-T1^R^ *E coli* (71). Cultures were then grown in LB media at 37 °C to an OD_600_ of ∼0.8 and induced with 1 mM IPTG for 3 hours at 37 °C, harvested by centrifugation, resuspended in Lysis Buffer (50 mM Tris/HCl (pH 8.0 at 20 °C), 500 mM KCl, 10 mM Imidazole/NaOH (pH 7.0 at 20 °C), 5 mM MgCl_2_, 5 mM BME, 1 mM PMSF), snap frozen in liquid nitrogen and stored at −80 °C. Thawed cells were lysed by extrusion (Emulsiflex C5, Avestin Inc), and centrifuged at 17,000 RPM in a JA-20 rotor at 4 °C for 40 min. Lysate was applied to Nickel Sepharose FF beads (GE) in batch mode and beads were collected by low-speed centrifugation. The beads were washed with 50 mM Tris/HCl (pH 8.0 at 20 °C), 500 mM KCl, 10 mM Imidazole/NaOH (pH 7.0 at 20 °C), 5 mM MgCl_2_ and then with 50 mM Tris/HCl (pH 8.0 at 20 °C), 500 mM KCl, 45 mM Imidazole/NaOH (pH 7.0 at 20 °C), 5 mM MgCl_2_ before eluting with 50 mM Tris/HCl (pH 8.0 at 20 °C), 500 mM KCl, 250 mM Imidazole/NaOH (pH 7.0 at 20 °C), 5 mM MgCl_2_. Elution fractions were incubated at 4 °C to allow excess SEPT2 to precipitate, which was removed by centrifugation at 3724*g* for 10 min in a SX4750 swinging bucket rotor (Beckman). SEPT2/6/7 complexes were then polymerized by overnight dialysis against 100 mM KCl, 1 mM MgCl_2_, 10 mM HEPES/NaOH (pH 7.0 at 20 °C), 0.5 mM EGTA, and 1 mM DTT. Filaments were collected by centrifuging at 55,000 RPM in a Ti70 rotor for 2 h, at 4 °C. The septin filament pellet was resuspended in 500 mM KCl, 50 mM Tris/HCl (pH 8.0 at 20 °C), 5 mM MgCl_2_, 1 mM DTT, and centrifuged again at 21,180 RCF for 10 min to remove residual uncomplexed SEPT2. SDS-PAGE analysis was used to check for purity, the sample was quantified by UV absorbance, glycerol was added to 20% w/v and ∼100 μl volumes were snap frozen in liquid nitrogen and stored at −80 °C.

#### mCherry-SEPT2/6/7

Two plasmids, pET11 His-(TEV recognition sequence)-SEPT2-mCherry and pCDF-SEPT6 MBP-(TEV recognition sequence)-SEPT7, were co-transformed into BL21(DE3)-T1^R^ cells and plated onto LB-agar plates containing ampicillin and spectinomycin. Protein expression, lysing, and centrifugation were performed as described above in WT SEPT2/6/7. Clarified lysates were applied to Amylose High-Flow beads (New England Biolabs) and washed extensively with AmyWB (25 mM Tris pH 8, 500 mM NaCl, 2 mM MgCl_2_) and eluted with AmyEB (25 mM Tris 8, 500 mM NaCl, 30 mM Maltose). The highest concentration amylose elution fractions were pooled and applied to Nickel Sepharose FF beads (GE) in batch mode and beads were collected by low-speed centrifugation. The beads were washed with 50 mM Tris pH 8, 500 mM NaCl, 10 mM Imidazole pH 7, 5 mM MgCl_2_ then with 50 mM Tris pH 8, 500 mM NaCl, 5 mM MgCl_2_, 45 mM Imidazole 7. Septins were eluted with 50 mM Tris pH 8, 500 mM NaCl, 5 mM MgCl_2_, 250 mM Imidazole pH 7. Fractions with the highest concentrations were pooled and MBP and His tags were removed by overnight digestion with TEV protease overnight. Septins were then further purified on a HiLoad 26/600 Superdex 200 PG column. Gel filtration buffer used was 500 mM NaCl, 25 mM Tris pH 8, 2 mM MgCl_2_, and 1 mM DTT. SDS-PAGE analysis was used to check purity and septins were quantified by UV absorbance. Glycerol was added to 20% w/v and ∼100 μl volumes were snap-frozen in liquid nitrogen and stored at −80 °C.

#### SEPT2Δα_0_/6/7

Two plasmids, pET11 His-(TEV recognition sequence)-SEPT2Δα_0_ and pCDF SEPT6-MBP-(TEV recognition sequence)-SEPT7, were transformed and expressed as described for SEPT2/6/7 WT. Thawed pellets were lysed *via* sonication and centrifuged as described above. Clarified lysate was applied to Amylose high flow beads (New England Biolabs) and washed with 500 mM NaCl, 50 mM Tris pH 8, 1 mM DTT, 5 mM MgCl_2,_ and eluted with 500 mM NaCl, 50 mM Tris pH 8, 1 mM DTT, 5 mM MgCl2, 30 mM maltose. Pooled samples were incubated with TEV protease overnight to release His_6_ and MBP fusions and the next day were applied to a HiLoad 26/600 Superdex 200pg column. The gel filtration buffer used was 500 mM NaCl, 20 mM Tris pH 8, 2 mM MgCl_2_, and 1 mM DTT. SDS-PAGE analysis was used to check purity and septins were quantified by UV absorbance. Glycerol was added to 20% w/v and ∼100 μl volumes were snap-frozen in liquid nitrogen and stored at −80 °C.

### Other proteins

The plasmid for profilin expression as a His_6_-tagged fusion from pET15b (Novagen) was a gift from Michael Rosen. It was expressed in BL21(DE3)T1^R^ by growth to an OD_600_ of 0.8 and induced with 1 mM IPTG. Induction proceeded for 16 h at 20 °C. Profilin was purified using nickel agarose affinity chromatography followed by Superdex 75pg 26/600 gel filtration chromatography. The pET11 plasmid for untagged Cdc42 was a gift from Michael Rosen (72). Cdc42 was expressed in BL21(DE3) T1^R^ *E coli* and then purified using DEAE Sepharose FF followed by SOURCE15Q anion exchange chromatography and gel filtration using Superdex75pg, all in the presence of 2 mM MgCl_2_. GMPPNP nucleotide was exchanged into Cdc42 by adding 5 mM EDTA and 690 μM GMPPNP, incubating for 2 h at 4 °C, then quenching with 10 mM Magnesium Chloride and finally buffer exchanging into “1× KMEI” (50 mM Potassium Chloride, 10 mM Imidazole pH 7.0 at 20 °C, 1 mM EGTA, and 1 mM Magnesium Chloride) using a Sephadex G-25 desalting column.

### Co-pelleting assays

#### Cdc42EP3 with septin filaments

Septin filaments were produced by dialysis of SEPT2/6/7 hexamers against a low salt dialysis buffer (LSDB: 100 mM KCl, 1 mM MgCl_2_, 10 mM HEPES/NaOH (pH 7.0 at 20 °C), 1 mM DTT), using a 14 kDa cutoff dialysis tubing. 100 μl samples were prepared with 7 μM septin filaments, 1 μM Cdc42EP3 or both, with remaining volume made up with dialysis buffer. Samples were incubated on ice for 1 h and then centrifuged at 21,130*g* (15,000 RPM) for 15 min 30 μl was removed from the top as the ‘supernatant,’ then an additional 55 μl was removed and discarded, leaving the ‘pellet’ fraction. The pellet was resuspended in 85 μl of 500 mM KCl, 50 mM Tris/HCl pH 8.0, 5 mM MgCl_2_, 1 mM DTT. Samples were analyzed by SDS-PAGE using a fixed 12% Criterion TGX Stain-Free 26 well precast gel (Bio-Rad) and imaged using Stain-Free and Coomassie Brilliant Blue imaging.

#### Cdc42EP3 with actin filaments

Actin filaments were prepared by dilution of actin monomers with a buffer concentrate that added a final composition of 12.5 μM actin in “1× KMEI” followed by incubation for 1 hour at 20 °C. 200 μl of Cdc42EP3 was centrifuged at 100,000 for 1 h to remove any aggregated material and the top 150 μl was used for the assays. 250 μl samples containing 2 μM Cdc42EP3, 10 μM actin filaments, or both were diluted in high-speed microfuge tubes (Beckman Coulter, #357448), with the remaining volume made up with 1× KMEI. Samples were incubated on ice for 40 min and then were spun at 100,000*g* (49,000 RPM in TLA 100.3 rotor) in a Beckman tabletop ultracentrifuge for 1 h at 4 °C. The top 100 μl was used as the ‘supernatant’ and the next 130 μl were discarded. The ∼10 μl remaining liquid with the pellet was resuspended in 230 μl Buffer G to break up actin filaments and then 240 μl of SDS-PAGE loading buffer (with SDS and BME) was added to ensure the breakdown of filaments. Samples were analyzed by SDS-PAGE using a fixed 12% Criterion TGX Stain-Free 26-well precast gel (Bio-Rad) and imaged using Stain-Free and Coomassie Brilliant Blue imaging.

#### Actin and septin filaments

For the separately polymerized filaments experiment, septin filaments were produced by dialyzing 8.3 μM SEPT2/6/7 hexamers into 1× KMEI + 1 mM DTT buffer overnight. Actin filaments were produced by the addition of 10× KMEI to 1/10th the final volume to a solution of 30.8 μM actin monomers and incubating at 20 °C for 1 h. 250 μl samples were prepared with 3 μM septin filaments, 3 μM actin filaments, or the combination of the two diluting with 1× KMEI as necessary and incubated at 4 °C for 1 h. Samples were then processed for the SEPT2/6/7 and Cdc42EP3 co-sedimentation, scaling volumes appropriately.

For the co-polymerized experiment, samples were prepared with 3 μM SEPT2/6/7 hexamer 3 μM actin, or both, and diluted to final volume with 1× KMEI. Samples were dialyzed overnight against 1× KMEI with added 1 mM DTT and 200 μM ATP. Each sample was then processed for the SEPT2/6/7 and Cdc42EP3 co-sedimentation, scaling volumes appropriately.

#### Actin monomer with septin filaments

SEPT2/6/7 hexamers were polymerized by dialysis into LSDB (see above). Samples containing 1 μM septins, 1 μM actin, or both were prepared, using LSDB to dilute to their target volume (200 μl), and incubated on ice for 3 hours before centrifugation. Samples were then processed for the SEPT2/6/7 and Cdc42EP3 co-sedimentation. To keep actin in a monomeric state, a stock solution of 15 μM actin with 20 μM Latrunculin A (from an ethanol stock) was prepared.

#### SEPT2Δα_0_/6/7 and actin filaments

SEPT2Δα_0_/6/7 was dialyzed into 1× KMEI. The next day, septins were spun at 100,000*g* for 30 min to remove any filaments. Actin filaments were produced by the addition of 10× KMEI to 1/10th the final volume to a solution of 16 μM actin monomers and incubating at 20 °C for 1 hour, generating a 14 μM stock of actin filaments. F-actin and septins were mixed in a 200 μl sample, brought up to volume with 1× KMEI, and incubated for 1 hour on ice. The final concentration of actin was 6 μM and septins was 2 μM. Samples were spun at 100,000*g* for 30 min. Samples were then processed for the SEPT2/6/7 and Cdc42EP3 co-sedimentation, scaling volumes appropriately.

### Fluorescence anisotropy assays

Most fluorescence anisotropy-based binding assays were performed with 100 μl volumes in black, polystyrene, half-area, 96-well plates (Corning, #3993). Each assay had 20 nM TMR labeled Cdc42EP3 and 1 mg/ml BSA, plus the indicated concentrations of actin, septins, or Cdc42. To generate septin filaments, septins were dialyzed overnight into 1× KMEI. For actin binding assays, actin was first polymerized by adding 10× KMEI to 1/10th of the final volume. Monomeric actin was prepared by adding Latrunculin to 20 μM in a 13 μM actin stock, after which 1/10th volume of 10× KMEI was added. For Cdc42EP3 binding to all these proteins, increasing amounts of the target protein were titrated into independently prepared wells, controlling for changes in volume by the addition of an appropriate filler buffer to maintain consistent solution conditions. The only exception was the measurement of Cdc42EP3 residues 1 to 21 to actin filaments, which was modified to allow higher concentrations of actin to be added. Here the assays were performed at a concentration of 5 nM labeled Cdc42EP3 residues 1 to 21, and the assays were performed at a total volume of 22 μl in a low volume 384-well plate.

Fluorescence anisotropy was acquired using a Tecan Spark multimode plate reader with the “enhanced fluorescence” package. The excitation wavelength was 523 nm with 10 nm bandpass and the emission wavelength was set to 579 nm with a 30 nm bandpass, with a 560 nm cutoff dichroic mirror between the excitation and emission light-paths. Each sample was read 10 independent times, with 200 flashes per read, and fluorescence anisotropy values were automatically generated by the SparkControl software. These data were averaged. Each condition was set up with 3 to 4 independent replicates, which were averaged prior to plotting. Note that the G-factor was not adjusted run to run, which resulted in some variability in the exact F.A. values between experiments.

### Actin polymerization kinetics with SEPT2/6/7 and Cdc42EP3

Pyrene actin polymerization assays were performed with 4 μM total actin (5% pyrene labeled) and 5 μM profilin (main figures). Septin filaments and Cdc42EP3 were prepared by dialyzing Cdc42EP3 and SEPT2/6/7 hexamers into 1× KMEI buffer plus 1 mM DTT. Profilin-actin mixes were prepared incubating monomeric actin in buffer G with profilin for 10 min. 10 E/1M buffer (10 mM EGTA pH 8, 1 mM MgCl_2_) was then added to the profilin-actin mix and Buffer G-Mg (2 mM Tris-HCl pH 8, 200 μM ATP, 0.5 mM DTT, 0.1 mM MgCl_2_, 1 mM sodium azide) was added to a final volume of 50 μl. A second mixture containing 10× KMEI, Cdc42EP3, and/or SEPT2/6/7 filaments as indicated, and brought up to a final volume with 1× KMEI. To start polymerization, these two solutions were mixed at a 1:1 ratio, placed in a fresh well of a 96-well half-area plate. Final SEPT2/6/7 and Cdc42EP3 concentrations in the indicated samples were 500 nM each. Fluorescence intensity acquisition was started as soon as possible after mixing, typically with a dead time of 70 to 130 s. Fluorescence intensity was recorded every 5 s, exciting at 365 nm, and 5 nm bandpass, and detecting emission at 407 nm with 5 nm bandpass, with a 410 nm dichroic mirror separating the excitation and emission light paths. Pyrene actin polymerization assays omitting profilin were performed with 2 μM actin (10% pyrene labeled) and measuring every 5 s but otherwise using the same method. Actin depolymerization assays were performed with 2 μM actin (10% pyrene labeled). Polymerization was set up as above but in a microfuge tube protected from light. After 2 hours of polymerization, depolymerization was then induced by the addition of Latrunculin A to 2.9 μM. The samples were then placed into the Tecan multimode plate reader and measured as described above, measuring every 1.7 s.

### Analytical gel filtration

Analytical gel filtration was performed using a Superdex 200 Increase 10/300 Gl column, with 500 μl loops for application. Chromatograms were monitored at 280 nm, 290 nm and 214 nm. The gel filtration column was calibrated using individual injections of the molecular weight standards RNAse A, conalbumin, aldolase, catalase, and thyroglobulin, as well as with a void volume standard of Blue Dextran 2000.

For the Cdc42EP3-actin complex gel filtration experiment, samples contained 10 μM Cdc42EP3, 10 μM Latrunculin A-actin, or both. For the Cdc42EP3-Cdc42(GMPPNP) and Cdc42EP3-Cdc42(GDP) binding experiments, samples were made up of 7 μM Cdc42EP3, 7 μM Cdc42, or both. We note that the exact tubing used differed between the calibrated experiments with actin and the Cdc42 binding experiments, so absolute elution volumes differ slightly between the experiments.

### Analytical ultracentrifugation

Wild type Cdc42EP3 was dialyzed into 1× KMEI overnight, quantified by UV absorption, and then diluted to 5 μM, to 3.75 μM, or to 1.67 μM with dialysis buffer. These samples were loaded into a sapphire windowed 1.2 cm thick two-channel centerpiece and placed into a An60-Ti rotor in an Optimal XL-I analytical centrifuge. The sedimentation velocity of all three samples was analyzed at 42,000 RPM at 20 °C for 8 h, monitoring sedimentation progress by the radial dependence of the absorption at 232 nm, scanning approximately every 4 minutes.

### Imaging

#### Static imaging of septin-actin filaments

Total internal reflection fluorescence microscopy Imaging was performed on samples prepared with 400 nM SEPT2-mCherry/6/7 (stock is 1.45 μM), 2 μM Actin (7% Oregon Green labeled, 5% biotinylated with actin stock mix being at 10 μM), and/or 150 nM 647-Cdc42EP3 (stock 658 nM), in single, pairwise combinations or a combination of all three materials. Samples were prepared and incubated at room temperature for at least 1 h to allow for polymerization. For samples where one or two proteins are left out, the buffer the missing proteins are in was added to ensure consistent salt and buffer conditions throughout all the samples. Actin was in G buffer (2 mM Tris pH 8.0, 200 μM CaCl_2_, 200 μM ATP, 1 mM Sodium Azide, 0.5 mM DTT). Septins were in 300 mM NaCl, 20 mM Tris pH 8, 1.6 mM MgCl_2_, 800 nM DTT, 20% w/v glycerol. Cdc42EP3 was in 120 mM NaCl, 8 mM HEPES pH 7, 800 nM DTT, 20% w/v glycerol. Before adding samples to flow chambers, oxygen scavenging mix was added to prevent photobleaching. To prepare this mix, first made stocks of 20 mg/ml glucose oxidase, 450 mg/ml d-glucose and 3.5 mg/ml catalase in 1× BRB80 buffer (80 mM PIPES pH 6.9, 1 mM MgCl_2_, 1 mM EGTA). Then a mixture containing 10% v/v of each, plus 280 mM βME, and brought up to volume with 1× KMEI. Then diluted this 1:20 with each sample. Samples were then loaded into flow chambers for imaging.

Flow chambers were prepared as follows. Square glass coverslips (18 mm and 25 mm #1.5, VWR Scientific) were cleaned by sonication in a series of cleaning solutions: 1% Micro-90 detergent, 100% ethanol, and 1 M potassium hydroxide, washing with ultra-pure water following each step. Dried coverslips were assembled into flow chambers using double-sided tape (∼80 μm thick). Using surface tension and wicking, the flow chambers were initially wet with ultrapure water. Then 1 mg/ml biotinylated BSA was flowed in and incubated for 10 min at room temperature. The chamber was washed twice with dialysis buffer to remove unbound BSA. 0.1 mg/ml streptavidin in dialysis buffer was flowed in and bound for 10 min before washing twice with two washes of dialysis buffer. Finally, dialyzed septin, actin, and/or Cdc42EP3 samples were flowed into the chamber, and the chamber was sealed with nail polish. Samples were imaged within 3 h of preparation using a GE DeltaVision OMX V4 with Olympus 60x 1.49 NA TIRF objective. Oregon Green actin was excited with a 488 nm laser and detected by emission at 528 nm with a 48 nm bandpass; mCherry was excited with a 568 nm laser and detected by emission at 609 nm with a 37 nm bandpass; Alexa 647-Cdc42EP3 was excited with a 642 nm laser and detected by emission at 683 nm with a 40 nm bandpass.

#### Kinetic TIRF imaging

Glass coverslips (60 × 24 mm; Thermo Fisher Scientific) were first cleaned by incubating with 1% Hellmanex III cleaning solution (Hellma Analytics) for 1 h at 60 °C. Coverslips were then rinsed thoroughly with MQ water and subsequently incubated in 180-proof ethanol for 5 min. Coverslips were then rinsed again in MQ water and incubated in a beaker filled with 0.1 M KOH for 10 min. Coverslips were then washed one more time with MQ water and dried with an N_2_ stream. The cleaned, dried coverslips were then coated with 40 mg/ml methoxy-polyethylene glycol (PEG)-silane MW 2000 and 8 mg/ml biotin-PEG-silane MW 3400 (Laysan Bio) in 80% ethanol, pH 2.0, by incubation overnight at room temperature. The next day, coverslips were rinsed with MQ water, dried with an N_2_ stream and stored in 50 ml conical tubes at −80 °C for up to 6 months. Before use in TIRF experiments, flow cells were assembled by rinsing PEG-coated coverslips with water, drying with N_2_, and adhering them to μ-Slide VI 0.1 (0.1 × 17 × 1 mm) flow chambers (Ibidi) with double-sided tape (2.5 cm × 2 mm × 120 μm) and 5-min epoxy resin (Devcon). Before each reaction, the flow cell was incubated for 1 min with 1% bovine serum albumin (BSA) in HEK buffer, and then equilibrated with TIRF buffer (50 mM imidazole, pH 7.4, 50 mM KCl, 1 mM MgCl_2_, 1 mM ethylene glycol-bis(β-aminoethyl ether)-N,N,N′,N′-tetraacetic acid (EGTA), 0.2 mM ATP, 10 mM DTT, 15 mM glucose, 20 μg/ml catalase, 100 μg/ml glucose oxidase) plus 0.5% methylcellulose (4000 cP). Finally, actin and other proteins (as specified in figure captions) have flowed into the chamber.

Time-lapse TIRF microscopy was performed using a Nikon-Ti200 inverted microscope (Nikon Instruments) equipped with a MLC400 Monolithic Laser Combiner (Agilent Technology), a TIRF-objective with a numerical aperture of 1.49 (Nikon Instruments), and an EMCCD camera (Andor iXon). The pixel size corresponded to 0.14 μm × 0.14 μm. All reactions were imaged once per minute for 30 minutes, acquired using 100 ms exposure times and imaging in two (actin + SEPT2/6/7) or three (actin + SEPT2/6/7 + Cdc42EP3) phases: (a – Alexa488 Actin) Excite with 488 nm laser, detecting at 525 nm with 50 nm bandpass, (b – mCherry SEPT2) Excite with 561 nm laser, detect emission at 605 nm with 55 nm bandpass, (c – Alexa647-Cdc42EP3) Excite 640 nm laser, detect emission at 700 nm with 75 nm bandpass. During recordings, focus was maintained using the Perfect Focus System (Nikon Instruments).

To visualize septin-actin co-assembly by live TIRF imaging, we first dialyzed 2 μM SEPT2/6/7 (10% SEPT2-mCherry/SEPT6/SEPT7-StrepII, 90% unlabeled SEPT2/6/7) –with or without 400 nM Alexa647-Cdc42EP3 –against 10 mM PIPES pH 6.9, 50 mM KCl, 1 mM MgCl2, 1 mM EGTA, 0.5 mM DTT for 2 h. 4.4 μM actin (10% Alexa488-labeled, 0.25% biotinylated) with 22 μM profilin was dialyzed against Buffer G. Next 1/9th volume of 10× KMEI was added to the actin to bring the concentration to 4 μM and initiate assembly. This was immediately mixed 1-1 with the dialyzed septins and applied to the flow chambers. The final concentrations were 2 μM actin, 10 μM profilin, 1 μM SEPT2/6/7, and 200 nM Cdc42EP3 (when present). The dead time between the addition of KMEI to actin and the first image was approximately 60s and was accounted for in the timing of filament appearance.

### Bioinformatics

The protein sequence for human Cdc42EP3 was obtained from NCBI protein, #NP_006440. A collection of 206 Cdc42EP3 sequences (#ENSG00000163171) was obtained from the GeneTree database at Ensembl (73) and re-aligned using Clustal Omega (74, 75, 76). The aligned sequences were used to compute Shannon Entropy (54) for each alignment column with a residue present in the human sequence using the ProDy package in Python 3 (53). Shannon Entropy values were smoothed with a seven-residue rolling average centered on the residue of interest, padding the end values by reflection. ‘Named region’ boundaries and mutation locations are previously described (22, 36, 37).

## Quantification and Statistical analysis

### Fluorescence anisotropy

The dose response of the fluorescence anisotropy was fit to:

$$r_{obs}=r_{bound}f_{bound}+r_{free}\left(1-f_{bound}\right)$$

Where *f*_*bound*_ follows a single site binding isotherm:

$$f_{bound}=\frac{\left(\left(K_{D}+L_{total}+P_{total}\right)-\sqrt{{\left(K_{D}+L_{total}+P_{total}\right)}^{2}-4L_{total}P_{total}}\right)}{2P_{total}}$$

Fitting was accomplished using the “curve_fit” method from SciPy in Python 3.0 (77) with the default Levenberg-Marquart optimization method, floating the parameters *r*_*bound*_, *r*_*free*_ and *K*_*D*_. In this equation, *r*_*obs*_ is the experimentally observed value of FA for the TMR labeled Cdc42EP3, *r*_*bound*_ is the FA of a ligand-saturated TMR-labeled Cdc42EP3 and *r*_*free*_ is the FA of an unliganded TMR-labeled Cdc42EP3. *K*_*D*_ is the binding dissociation constant, *L*_*total*_ is the added titrant (actin, septin, *etc.*) concentration and *P*_*total*_ is the labeled Cdc42EP3 (20 nM). Data used in fitting was the average of 3 to 4 replicates for each concentration. Reported errors are the one-sigma error estimate from the parameter covariance matrix.

### Quantification of pelleted fraction

For filament spin-down assays, the pelleted fraction was calculated by integrating the band intensity from ‘Stain-Free’ fluorescent gels, for the supernatant and pelleted fractions. Rectangular regions of interest (ROI) of equal size and large enough to fit the largest band in a group were created in the ROI manager in NIH ImageJ/FIJI (78). The background was estimated in identically sized ROIs centered above and below the band of interest, avoiding any other proteins that may be present. The integrated intensity was collected using the ‘Measurement..’ function in ImageJ/FIJI. Above and below band background intensities were averaged and subtracted from the raw band intensity. Then the ‘pelleted fraction’ was calculated as the fluorescence intensity in the pellet fraction divided by the sum of the pelleted and supernatant fractions. This process was repeated for each supernatant/pellet band pair.

### Actin polymerization assays

From the kinetic progress curve, data was quantified by the time to 50% polymerization (*t*_*50*_) using a previously described method (70). Briefly, the initial and saturating intensities were determined, averaging intensities locally. Then points near the 50% intensity value were determined automatically and used to infer the time of 50% intensity by linear extrapolation. Reported values of include an offset for the deadtime of the reaction.

### Analytical gel filtration

Peak maxima were determined by fitting an ∼0.3 ml interval near the peak maxima to a parabola and determining the maxima from that function. From the peak retentions (*V*_*e*_), *K*_*AV*_ were calculated using:

$$K_{AV}=\frac{V_{e}-V_{0}}{V_{total}-V_{0}}$$

The void volume (*V*_*0*_) was estimated from the Blue Dextran 2000 standard to be 8.53 ml and the total volume (*V*_*total*_) was estimated to be 20.50 ml. *K*_*AV*_ was then plotted and fit as a function of *log*_*10*_ of the standard molecular weights. From this linear fit, we inferred the apparent molecular weight of Cdc42EP3, assuming a compact globular protein (which the standards are reasonable approximations of).

### Analytical ultracentrifugation

Data was loaded into SEDFIT (79) corrected for timing irregularities, and fit to *c*(*s*) and *c*(*M*) distributions. *c*(*s*) fitting optimized frictional ratio, meniscus position, and sample bottom position. Additionally, time-independent noise was removed from the data. Molecular weight was inferred by integrating the dominant peak in *c*(*M*) distribution. Buffer density and viscosity were approximated using 50 mM potassium chloride, 10 mM Tris, 1 mM magnesium chloride, and 1 mM EDTA in the Buffer Management database of UltraScan-III (80). c(*s*) and data-fit-residual plots were prepared using GUSSI (81).

### Determination of the number of actin filaments per septin bundle

Images were analyzed using NIH ImageJ/FIJI (78). For each image, a line scan of the filament bundle was taken at five separate points along the length of the bundle. Line scans were also taken at the center of five individual actin filaments near the bundle, and five points in the background in close proximity to the bundle. The average background intensity was calculated and subtracted from the intensities of the individual filaments as well as the intensity of the bundle at the different points, correcting for line scan lengths. The average background subtracted intensity for the individual actin filaments and bundle was calculated and the average intensity of the bundle was divided by the average intensity of the individual filament to estimate the number of filaments per bundle. This was done for six images for both the septin-actin samples and septin-actin-Cdc42EP3 samples. Quantitation was assessed using a Student’s *t* test as described below.

### Kinetic analysis of imaged filament nucleation and growth

All recorded movies were analyzed using NIH ImageJ/FIJI (78). Hyperstack images were opened, background subtracted using the ‘rolling ball’ method with a 50-pixel radius. The contrast was then set manually for each channel to match across fields of view. The FilamentFinder2 plugin (82) was used to track the actin filament number and length in the actin channel (Alexa488), with manual interventions. Where entire filaments could be unambiguously tracked for at least five frames (determined by manual inspection), filaments were tracked frame to frame with refinement using the active contour method. Where filament trace refinement was stuck in local minima, filament trajectories were manually edited and reoptimized. Where filaments crossed image edges, were lost in clusters or otherwise prevented unambiguous assignment over time, filaments were manually traced but not tracked over time. Thus, filament counts were accurate while ambiguous filaments were excluded from length calculations. Finally, where initial 1 to 2 frames showed anomalously rapid elongation consistent with a preformed filament falling into the TIRF illumination zone, initial frames were excluded. Tracing of every single filament in each field of view continued until the field of view was too crowded to accurately trace filaments. Filament trajectories (‘snakes’) were then saved and imported into a JuPyTer Lab python script for filament number counting, filament length calculations, growth, and nucleation rate calculations, and figure generation using SciPy, matplotlib, and Seaborn (77, 83, 84). Filament length was determined from the sum of segment lengths from active contour-optimized ‘snakes’. ‘Apparent Nucleation Rate’ was quantified by fitting the filament count over time to a straight line and reporting the slope.

### Statistical analysis

Statistical tests were performed in Python using the “SciPy” package for Student’s *t* test and 1-way ANOVA test (77), using the “statsmodels” package for Tukey-HSD tests (85). For each test, the critical *p*-values were defined as: ‘n.s.’ means *p* > 0.05, ‘∗’ means *p* < 0.05, ‘∗∗’ means *p* < 0.01, and ‘∗∗∗’ means *p* < 0.001. Nonlinear fitting of binding isotherms was accomplished using the curve_fit method in SciPy. Reported errors from fits are from the single parameter error estimates from curve_fit. Plotting was performed using the matplotlib (83) and seaborn (84) packages.

## Data availability

Further information and requests for resources and reagents should be directed to and will be fulfilled by the lead contact, Shae Padrick (sbp59@drexel.edu). Plasmids generated in this study are available upon request from the lead contact. Raw data and python script code used for analysis and figure generation are available on request from the lead contact.

Plasmid resource for expression of septin and Cdc42EP3 materials, raw data and processing scripts are available on request from the corresponding author.

## Supporting information

This article contains supporting information.

## Conflicts of interest

The authors declare that they have no conflicts of interest with the contents of this article.
