# Supplementary material for: Cdc42EP3-bound septin scaffolds promote actin polymerization
Source: J Biol Chem. 2025 Feb 18;301(3):108325. doi: 10.1016/j.jbc.2025.108325 (PMC11952830; doi:10.1016/j.jbc.2025.108325)
Supplement: Supporting Information [file mmc1.pdf]

Supporting Information

For Tomasso et al "Cdc42EP3-bound septin scaffolds promote actin polymerization"

One supplementary table and three supplementary figures

Supporting Table S1

Table of constructs

Construct	Parent vector	Insert	Notes
Cdc42EP3	pMAL-C2-(TEV)	Cdc42EP3<br>C-terminal His6	Synthetic codon optimized (for E. coli expression) sequence to match NCBI protein sequence: NP_006440
Cdc42EP3 C145A	pMAL-C2-(TEV)	Cdc42EP3<br>C-terminal His6<br>C145A	C211 remains as unique cysteine for labeling
Cdc42EP3 C145A "KLP→AAA"	pMAL-C2-(TEV)	Cdc42EP3<br>C-terminal His6<br>C145A K139A<br>L140A P141A	
Cdc42EP3 C145A "IS→AA"	pMAL-C2-(TEV)	Cdc42EP3<br>C-terminal His6<br>C145A I56A S57A	
Cdc42EP3 C145A, C211A, Cys0	pMAL-C2-(TEV)	Cdc42EP3<br>C-terminal His6<br>C145A C211A	Cys0 is a cysteine added at the N-terminus of the Cdc42EP3 sequence
CRIB/BH1	pMAL-C2-(TEV)	Cdc42EP3 residues 1-83, added Cys0 for labelling	
Cdc42EP3 1-21	pMAL-C2-(TEV)	Cdc42EP3 residues 1-21, added Cys0 for labeling	
Cdc42EP3 C145A C211A C-term TrpCys	pMAL-C2-(TEV)	Cdc42EP3<br>C-terminal His6<br>C145A C211A	Trp-Cys added to end of C-term His6 for labelling
ΔCRIB-BH1	pMAL-C2-(TEV)	Cdc42EP3 residues 74-254, with C-term His6	C-terminal Cys added for labeling
SEPT2-mCherry	pMAL-C2-(TEV)	SEPT2 with N-terminal MBP fusion (TEV cleavable) and C-terminal mCherry tag	Reference sequence for SEPT2: NP_001153189

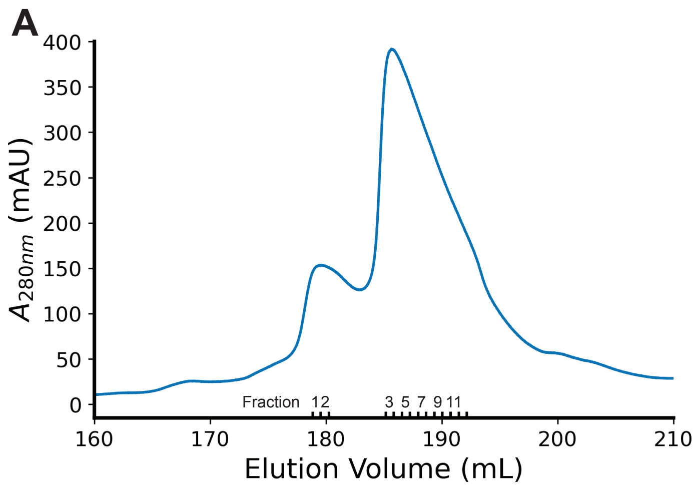

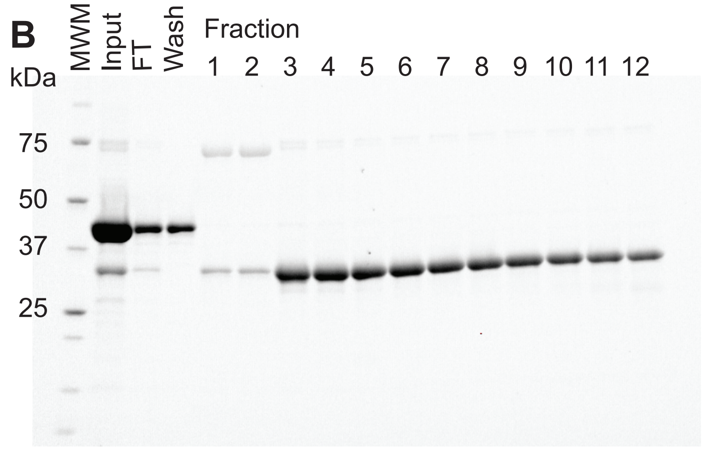

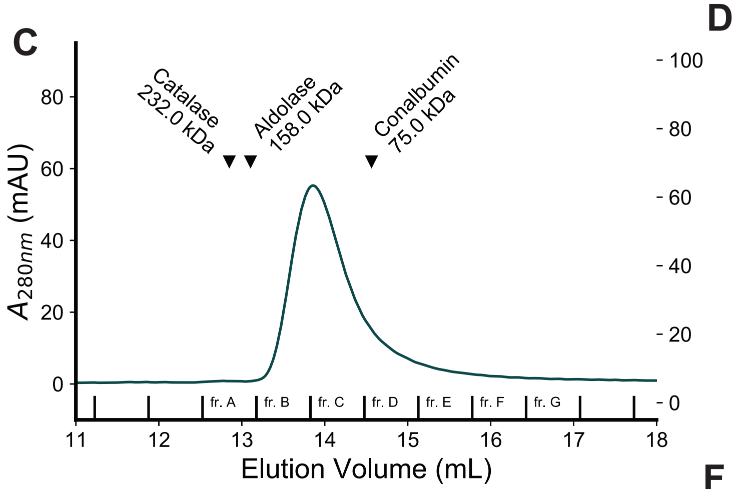

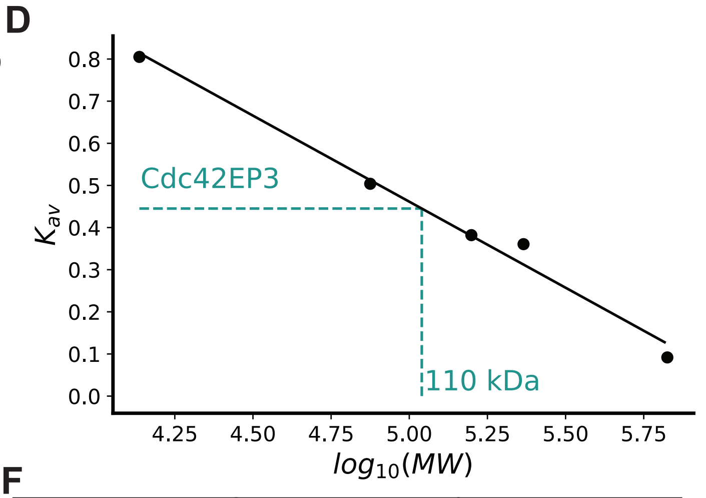

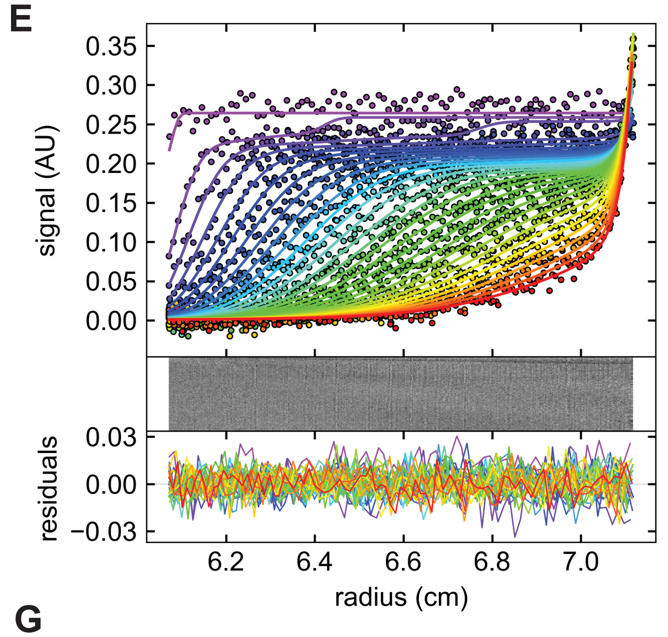

F

Cdc42EP3 concentration	Frictional Ratio	Mass
5 µM	1.51	24.2 ± 2.6 kDa
3.75 µM	1.66	27.1 ± 3.5 kDa
1.67 µM	1.50	23.2 ± 2.3 kDa

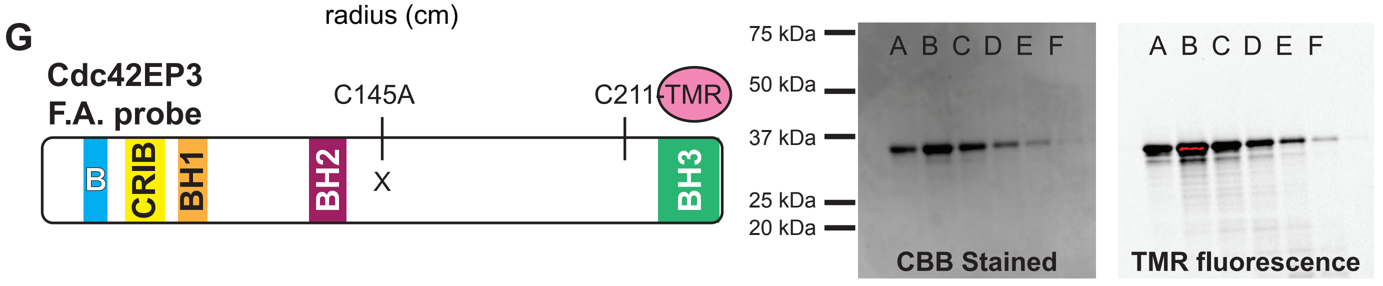

Supplemental Figure S1

Figure S1: Purification and hydrodynamic characterization of recombinant Cdc42EP3

A) Chromatogram (A_{280}) of Cdc42EP3 eluting off MonoS 5/50 column. B) SDS-PAGE analysis of MonoS fractions, fractions are numbered as shown in panel (A). C) Gel filtration chromatogram of Cdc42EP3. Elution volumes of protein standards and their molecular weights are given near the top. D) Elution volumes of protein standards were used to calculate estimated molecular weight of Cdc42EP3. E) SV-AUC was performed on purified Cdc42EP3 and fit to a *c*(*s*) model. Circles are every third data point from every fifth scan analyzed. The colored lines indicate the fit to data from *c*(*s*) analysis. Earlier time points are indicated by purple to blue, while later time points are indicated by yellow to red. F) Table showing the frictional ratio and apparent mass of the samples determined by AUC for the three different concentrations of Cdc42EP3. G) SDS-PAGE analysis of TMR labeled Cdc42EP3, imaged using coomassie brilliant blue staining (CBB) (left) or using TMR fluorescence (right). Red pixel are overloaded.

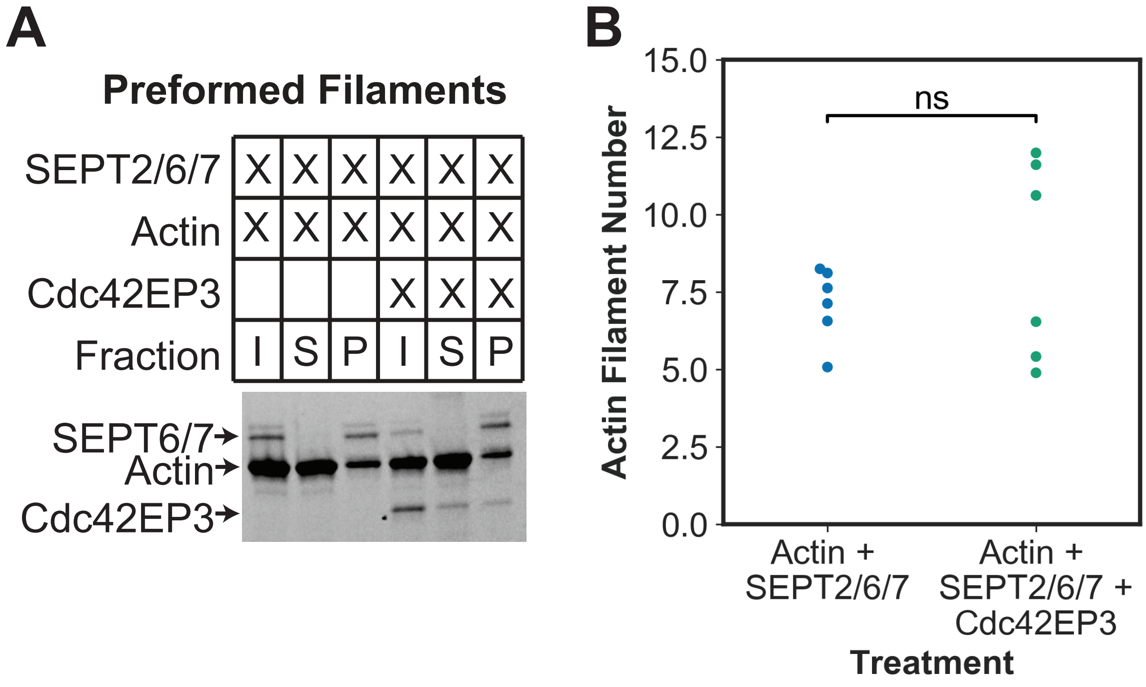

Supplemental Figure S2

Figure S2: Cdc42EP3 does not impact SEPT2/6/7-actin association

A) Septins and actin were co-polymerized together with or without Cdc42EP3 via dialysis overnight and centrifuged at 21,000**g* the following day. Input (I), Supernatant (S) and Pelleted (P) fractions were analyzed using SDS-PAGE and imaged using Stain-Free imaging. B) The approximate number of actin filaments per septin-associated actin bundle imaged using TIRF microscopy was quantified in septin-actin bundles with and without Cdc42EP3, n=6 for each group. ns= no significant difference found.

A

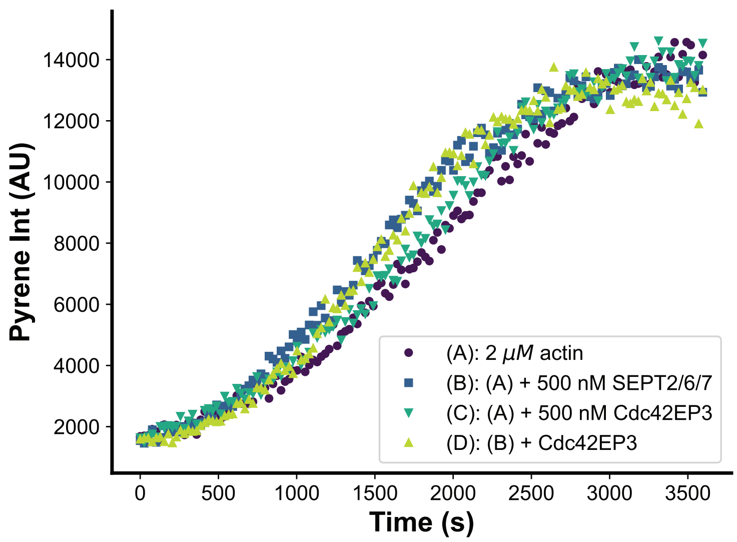

B

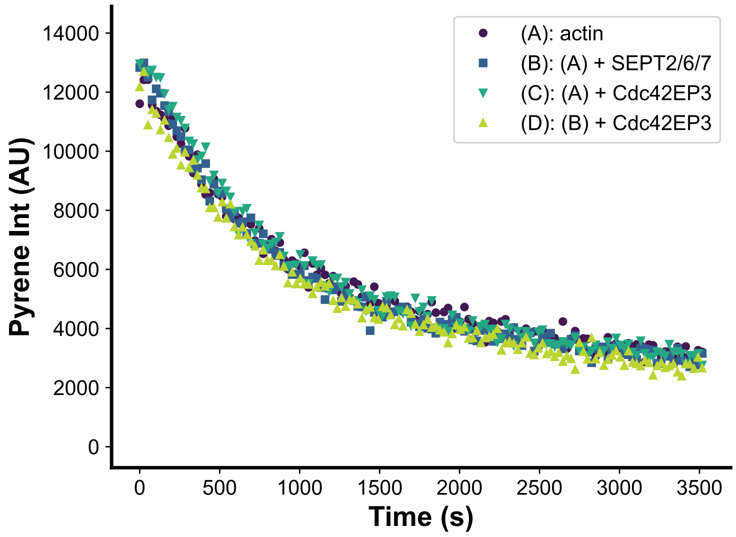

Supplemental Figure S3

Figure S3: Actin polymerization controls

A) Actin (10% pyrene labeled) was fully polymerized and then mixed with 500 nM Cdc42EP3, 500 nM SEPT2/6/7 filaments, or a mix of both, and incubated for two hours. Depolymerization was then initiated by the addition of excess Latrunculin A and pyrene intensity was tracked over time. B) Actin monomers (10% pyrene labeled) were mixed with polymerization buffer and 500 nM Cdc42EP3, 500 nM SEPT2/6/7 filaments, or a mixture of both. Actin polymerization was initiated by concurrent addition of salt and monitored by measuring fluorescence intensity of pyrene over time until polymerization was complete.
